# CCL5/RANTES signaling in inflammation dysregulation after mild traumatic brain injury

**DOI:** 10.1186/s12929-025-01203-0

**Published:** 2026-01-09

**Authors:** Man-Hau Ho, Yih-Jeng Tsai, Yu-Hsuan Lee, Yi-Chen Hsieh, Chia-Hung Yen, Jia-Yi Wang, Thierry Burnouf, Chia-Yen Chen, Wen-Cheng Lin, Yun Wang, Yung-Hsiao Chiang, Barry J. Hoffer, Szu-Yi Chou

**Affiliations:** 1 Ph.D. Program in Medical Neuroscience, College of Medical Science and Technology, Taipei Medical University and National Health Research Institute, 250 Wu-Xing Street, Taipei City, 11031 Taiwan; 2 Graduate Institute of Neural Regenerative Medicine, College of Medical Science and Technology, Taipei Medical University, Taipei, Taiwan 250 Wu-Xing Street, 11031; 3 Department of Otolaryngology Head and Neck Surgery, Shin Kong Wu Ho‐Su Memorial Hospital, Taipei, Taiwan; 4 School of Medicine, Fu Jen Catholic University, New Taipei City, Taiwan; 5 Taipei Neuroscience Institute, Taipei Medical University, Taipei, Taiwan; 6 Department of Neurosurgery, Shuang Ho Hospital, Taipei Medical University, New Taipei City, Taiwan; 7 Department of Biological Science and Technology, National Pingtung University of Science and Technology, Neipu, Pingtung, 91201 Taiwan; 8 Graduate Institute of Medical Sciences, College of Medicine, Taipei Medical University, Taipei, 11031 Taiwan; 9 Department of Neurosurgery, Taipei Medical University Hospital, Taipei, 11031 Taiwan; 10 Neuroscience Research Center, Taipei Medical University, Taipei, 11031 Taiwan; 11 International PhD Program in Biomedical Engineering, College of Biomedical Engineering, Taipei Medical University, Taipei, Taiwan; 12 Graduate Institute of Biomedical Materials and Tissue Engineering, College of Biomedical Engineering, Taipei Medical University, Taipei, Taiwan; 13 NeuroTMULille International Laboratory, Taipei Medical University, Taipei, Taiwan; 14 International PhD Program in Cell Therapy and Regeneration Medicine, College of Medicine, Taipei Medical University, Taipei, Taiwan; 15 Center for Neuropsychiatric Research, National Health Research Institutes, Zhunan, 350401 Miaoli County, Taiwan; 16 Department of Surgery, School of Medicine, College of Medicine, Taipei Medical University, Taipei, 11031 Taiwan; 17 Hoffer Consulting, Cleveland, OH USA; 18 Scientist Emeritus, National Institutes of Health, Bethesda, USA; 19 International Master Program in Medical Neuroscience, College of Medical Science and Technology, Taipei Medical University, Taipei, Taiwan

**Keywords:** CCL5, CCR5 signaling, Microglia, Mild traumatic brain injury, Oxidative stress

## Abstract

**Background:**

Mild traumatic brain injury (mTBI) is the most prevalent form of brain injury. Secondary damage following mTBI contributes to neuronal degeneration by promoting neuroinflammation, amyloid accumulation, and oxidative stress (OS). Microglia exhibit dual roles after injury, contributing to both pro-inflammatory (M1) and anti-inflammation/neuroprotective (M2) responses. Targeting microglial polarization may therefore represent a therapeutic strategy for mitigating secondary damage after TBI.

**Methods:**

A weight-drop mTBI model (30 g, 100 cm) was applied to both C57BL/6 (wild-type) and CCL5 knockout (CCL5-KO) mice. Microglial activation was assessed at 7-, 14-, 21-, and 28-days post-injury using RT-qPCR, immunohistochemistry, and western blotting. Oxidative stress in tissue was detected by Hydroxyprobe™ labeling, ROS detection, NADPH oxidase activity assay, and antioxidant expression. Recombinant CCL5 (rCCL5) was administered intranasally to evaluate its effect on post-injury inflammation. Cortical tissue was subjected to liquid chromatography-tandem mass spectrometry (LC-MS/MS) for proteomic profiling. In vitro, BV2 microglial cells were treated with H_2_O_2_ to model OS. The effects of rCCL5 on cell viability, inflammatory gene expression, and phagocytic activity were assessed via MTT assay, immunocytochemistry, flow cytometry, and RT-qPCR. Pharmacological inhibitors targeting CCR1, CCR3, and CCR5 were used to delineate receptor-specific signaling pathways.

**Results:**

rCCL5 significantly reduced oxidative stress in both neurons and microglia and enhanced expression of antioxidant enzymes such as GPX1, SOD1, and SOD2 in injured cortices. Proteomic analysis revealed upregulation of immune regulatory and phagocytosis-related pathways following rCCL5 treatment. In vitro, rCCL5 conferred cytoprotection against H_2_O_2_-induced cell death and promoted M2-like microglial polarization. Blockade of CCR5, but not CCR3, abrogated CCL5-induced M2 differentiation, whereas both CCR3 and CCR5 were required for enhanced phagocytosis. CCL5-induced NFATc2 activation was mediated primarily via CCR5.

**Conclusions:**

These findings demonstrate that CCL5 modulates microglial polarization and attenuates oxidative stress in the injured brain through a CCR5-dependent mechanism. Targeting the CCL5–CCR5 signaling axis may offer a promising therapeutic strategy for improving outcomes after mTBI.

**Supplementary Information:**

The online version contains supplementary material available at 10.1186/s12929-025-01203-0.

## Introduction

Brain trauma commonly results in structural damage, such as axonal injury and disruption of the blood–brain barrier (BBB), similar to what is observed in stroke. In the acute phase, damaged neurons release reactive oxygen species (ROS) and danger-associated molecular patterns (DAMPs), which trigger neuroinflammation and activate glial cells, perpetuating a deleterious inflammatory cascade [1]. Chronic inflammation contributes to synaptic loss, cognitive impairment, and long-term neurodegeneration [2, 3]. Oxidative stress (OS) and inflammatory responses are two major factors causing neuronal damage in traumatic brain injury (TBI), stroke, and other neurodegenerative disorders [4–6]. Immune responses to brain injury, especially from microglia, are associated with the subsequent development of neurodegeneration after TBI. Macrophages and microglia can migrate to the injury site when the BBB is broken after brain injury. Microglia remove cell debris in response to TBI; microglia also release deleterious substances including pro-inflammatory cytokines, additional ROS, and reactive nitrogen species (RNS), which exacerbate neuronal damage [7]. Free radical overload, increased inflammatory molecules, excessive excitotoxicity, and mitochondrial dysfunction in neurons lead to neurological dysfunction either transiently or permanently.

The balance and regulation of immune responses and the clearance of debris and ROS after injury are keys to neuron functional recovery. According to their activation states, microglia can be classified as “classically activated” microglia (M1 microglia) and “alternatively activated” microglia (M2 microglia). M1 microglia are pro-inflammatory which produce cytokines and lead to neural injury; in contrast, M2 microglia are anti-inflammatory, release neurotrophic factors, activate phagocytosis, and promote neural repair processes [8]. Many studies and drugs used to treat TBI target ROS generation or manipulate microglial activation. Studies targeting NADPH oxidase to reduce ROS generation [9, 10] or detoxification of oxygen metabolites by NAC (N-acetyl L-cysteine) [11, 12], a precursor of glutathione, show significant improvement in brain injury studies. Inhibiting M1 microglia by donepezil, an acetylcholinesterase inhibitor, decreased neuroinflammation and apoptosis as well as improved memory-cognitive function after TBI [13–15]. In addition, NAC treatment not only reduces cellular OS but also activates M2 microglia [16]. These findings suggest the importance of a delicate balance between ROS and M2 microglial activation during recovery processes after brain injury.

Chemokine CCL5 (C-C motif ligand 5) shows a protective role for neuronal damage, such as in stroke [17] and Alzheimer’s disease [18–21]. TBI shares many common pathological features with stroke and Alzheimer’s disease, such as BBB rupture, micro-bleeds, insulin resistance and memory dysfunction. We have shown that CCL5 is an essential factor in activating glutathione peroxidase 1 (GPX1) after brain injury. Both CCL5 and the GPX-1 precursor -NAC reduced neuronal OS, protected hippocampal neurons from death and promoted recovery of memory-cognition function in mice after mild brain injury [22]. We also documented that CCL5 promotes cortical neuronal restoration and facilitates motor and sensory function recovery following mild TBI (mTBI). Intranasal administration of CCL5 to both wild-type (WT) and CCL5-deficient mice enhanced neuronal recovery by promoting axonal regeneration and remyelination, likely through the activation of the mTOR, Rho, and Neuregulin/ErbB signaling pathways [23]. Conversely, studies with blockade of the CCL5 receptor—CCR5—using the FDA-approved antagonist Maraviroc, or employing neuron-specific CCR5 knockout mice, further supported neuroprotective effects in both stroke and controlled cortical impact (CCI)-induced TBI models [24]. Additionally, CCR5 expressed in peripheral immune cells also contributes to neuronal recovery after TBI. In aged TBI mice, CCR2/5 expression is upregulated, resulting in enhanced recruitment of inflammatory cells and activation of NOX2. Treatment with either the dual CCR2/5 antagonist Cenicriviroc or the CCR5 antagonist Maraviroc attenuated inflammation, oxidative stress, and BBB disruption, thereby promoting functional recovery [25, 26]. Together these findings suggest that targeting CCR5 is a promising therapeutic strategy for stroke and TBI. However, the paradoxical roles of CCL5 and its receptor CCR5 remain unclear.

In the current study, both C57BL/6 wild-type (WT) and CCL5-knockout (CCL5-KO) mice were subjected to mild brain injury using a closed-head weight-drop model. We investigated the role of CCL5 in regulating oxidative stress and microglial chemokine expression in the cortex following mTBI. Oxidative stress and neuronal injury were assessed by NADPH oxidase activity, Hypoxyprobe labeling, and Fluoro-Jade C (FJC) staining. The inflammatory status of the brain was analyzed via LC-MS/MS-based proteomic profiling and quantitative PCR of chemokine transcripts. To explore the cellular mechanisms underlying CCL5-mediated microglial polarization and chemokine release under oxidative stress, the murine microglial cell line BV2 was employed. CCL5 promoted M2-like polarization and suppressed M1-like microglia polarization. Intranasal administration of recombinant CCL5 to both WT and CCL5-KO mice reduced cortical oxidative stress and enhanced M2-associated microglial chemokine expression and phagocytic activity, partially through regulation of NFATc2 nuclear translocation. In contrast, CCR5 blockade with Maraviroc inhibited NFATc2 nuclear translocation and suppressed M2 microglial polarization and phagocytosis.

## Materials and methods

### Animals and weight-drop model of mild TBI

All animal procedures were approved by the Institutional Animal Care and Use Committee of Taipei Medical University (Protocol Nos. LAC-2021-0004, LAC-2021-0360, and LAC2022-0490). A total of 80 male C57BL/6 J mice were obtained from the National Laboratory Animal Center (NLAC), NARLabs, Taiwan. Additionally, 100 male CCL5 knockout mice (B6.129P2-Ccl5^tm1Hso^/J, Stock No: 005090) were used which originally purchased from The Jackson Laboratory (USA) and maintained at NLAC. Animals were housed in a temperature-controlled (25 °C) environment under a 12:12-h light/dark cycle with ad libitum access to food and water.

Mild traumatic brain injury was induced using a closed-head weight-drop model as previously described [22, 23, 27–30] with body temperature monitored and maintained at normal levels [22]. In rescue experiments, recombinant mouse CCL5/RANTES protein (478-MR-025, R&D Systems) was administered intranasally at a dose of 300 pg/g 30 min before injury following protocols from our previous studies or administered at a dose of 30 pg/g since post injury 3 days every 2 days for 28 days. The volume used for CCL5 administration was controlled within 25–35 μl, at a concentration of 300 pg/μl or 30 pg/μl, which falls within the standard range for PBS administration [22, 23].

### LC–MS/MS proteomic analysis

Cortical tissues were collected from three independent mice per group (sham, TBI, TBI with CCL5 pre-treatment, and CCL5 post-treatment) for LC-MS/MS analysis, as previously described. [23]. Identified proteins were subjected to functional annotation using DAVID Bioinformatics Resources 6.8 for Gene Ontology (GO) and Ingenuity Pathway Analysis (IPA, Qiagen) for canonical pathways, networks, and upstream regulator prediction. Two comparison groups were analyzed: (1) CCL5⁻/⁻ TBI + PBS vs. CCL5⁻/⁻ TBI + CCL5 pre-treatment, and (2) CCL5⁻/⁻ TBI + PBS vs. CCL5⁻/⁻ TBI + CCL5 post-treatment, as well as (3) CCL5-/- TBI + CCL5 pretreatment vs CCL5-/- TBI + CCL5 post-treatment. Proteins with fold change > 1.25 or < 0.75 and p-value < 0.05 (unpaired *t*-test) were considered significantly altered.

GO Analysis: Enrichment analysis was performed to identify overrepresented biological processes, molecular functions, and cellular components.

IPA Analysis: Selected protein identifiers and log₂ fold changes were uploaded to IPA (Qiagen) and analyzed using the Core Analysis tool (default settings). Pathways associated with immune activation, inflammation, and oxidative stress were highlighted.

### Basal ROS and NADPH oxidase activity assay

ROS production and NADPH oxidase activity in cortical tissues were assessed at 4-, 7-, 14-, and 28-days post-injury (dpi); sham groups served as baseline. ROS was measured in 200 µl of Krebs buffer (pH 7.4) containing 50 µM lucigenin (Sigma-Aldrich, 2315971) using the Triathler Multilabel Tester (425-004, HIDEX, Finland) and normalized to tissue weight (µg). For NADPH oxidase activity, 50 µM NADPH was added to the mixture and luminescence was recorded for 350 s. The area under the curve (AUC) was calculated. Methods used followed our previous publication [22].

### Immunohistochemistry, Fluoro-Jade C (FJC) staining, and hypoxyprobe labeling

Mice were perfused with 4% paraformaldehyde (Sigma, 158127) in 0.1 M phosphate buffer (PB) under anesthesia. Brain tissues were cryosectioned at 35 μm. Sections were incubated in 1% sodium borohydride (in 0.1 M PB) for 30 min, then blocked with 3% normal goat serum and 3% BSA (bovine serum albumin) in PBS containing 0.25% Triton X-100. Primary antibodies were applied overnight at 4 °C: anti-Iba1 (GeneTex, GTX100042, 1:500) and anti-NeuN (GeneTex, GTX30773, 1:200). Sections were washed and incubated with secondary antibodies: donkey anti-rabbit 568 (Invitrogen, A10042, 1:400) and donkey anti-mouse 647 (Invitrogen, A-31571, 1:400) for 60 min at room temperature. Sections were mounted with VECTASHIELD (Vector H1000). Controls consisted of omission of primary antibodies and blinded observers.

FJC staining was performed following the manufacturer’s protocol (TR-100-FJ, Biosensis). Hypoxia was detected using the Hypoxyprobe^™^ Plus Kit (Biosensis) as previously described [22]. For Sholl analysis, images of microglia were captured using an LSM 900 confocal microscope (Zeiss) with a 10 µm Z-stack consisting of 4 Z-steps. The resulting projection images were further analyzed using the Neuroanatomy plugin in Fiji/ImageJ (NIH, v1.54). Intersections per micrometer were measured from the center of the cell at 5 µm intervals up to 100 µm. DAPI (4',6-diamidino-2-phenylindole, Sigma, D9542) was used for nuclear staining.

### RNA extraction, reverse transcription, and qPCR

Total RNA was extracted using TRIzol reagent (Invitrogen, 15596018) and reverse transcribed using the High-Capacity cDNA Reverse Transcription Kit (Applied Biosystems, 4368813). Quantitative PCR was performed with iTaq Universal SYBR Green Supermix (Bio-Rad) on a StepOnePlus^™^ Real-Time PCR System (Applied Biosystems, 4376600). Expression levels were normalized to GAPDH and calculated as 2^-ΔCT.

Primer sequences used:

**Table:** 

Gene	Forward primer	Reverse primer	Product size
CCL5	TGCTGCTTTGCCTACCTC	CTTGAACCCACTTCTTCTCT	151 bp
GPX1	CGTTTGAGTCCCAACATCTC	CGTTCATCTCGGTGTAGTCC	199 bp
IL-1β	GCACTACAGGCTCCGAGATGAAC	TTGTCGTTGCTTGGTTCTCCTTGT	147 bp
IL-6	AGTTGCCTTCTTGGGACTGA	TCCACGATTTCCCAGAGAAC	159 bp
TNF-α	GGAACTGGCAGAAGAGGCACTC	GCAGGAATGAGAAGAGGCTGAGAC	89 bp
IL-10	GCTCTTACTGACTGGCATGAG	CGCAGCTCTAGGAGCATGTG	105 bp
Arg-1	AGTCTGGCAGTTGGAAGCAT	GGAGAAAGGACACAGGTTGC	142 bp
GAPDH	GTGTTCCTACCCCCAATGTGT	AGAGTGGGAGTTGCTGTTGAAG	176 bp

### BV2 cell culture and viability assay

BV2 cells were kindly provided by Dr. CT Hong (Shuang-Ho Hospital, Taiwan). BV2 cells were maintained in DMEM (Gibco, 12100061) supplemented with 10% fetal bovine serum (Gibco, 10437028) and 100 U/ml penicillin–streptomycin (Gibco, 151402122). Cells (5 × 10^5^) were seeded in 6-well plates. Prior to CCL5 and H_2_O_2_ stimulation, the culture medium was replaced with DMEM containing 2% FBS. Cells were then treated with CCL5 at concentrations of 0, 250, 500, or 750 pg/mL for 30 min, followed by treatment with H_2_O_2_ (0, 250, 500, or 750 µM; PanReac AppliChem, 131077) for 24 h to assess cell viability. For the viability assay, MTT (10 µg/mL; Invitrogen, M6494) was added 1 h before cell harvest. After incubation, 200 µL of DMSO was used to lyse the cells, and absorbance at 550 nm was measured using a microplate reader (iMark microplate absorbance reader, Bio-Rad). Data were normalized to the untreated control group.

### M1/M2 immunocytochemistry (ICC) analysis, flow cytometry analysis, and NFATc2 nuclear translocation assay

BV2 cells were seeded in 60-mm culture dishes at a density of 1 × 10^6^ cells per dish. On the following day, the culture medium was replaced with DMEM supplemented with 2% fetal bovine serum (FBS). Cells were pretreated with 1 µM Maraviroc (HY-13004, MCE), 100 nM SB-328437 (HY-103363, MCE), or a combination of both inhibitors for 30 min. Following pretreatment, cells were treated with recombinant CCL5 protein for 30 min and subsequently exposed to 500 µM hydrogen peroxide (H_2_O_2_) for 18 h to induce oxidative stress.

For M1/M2 ICC analysis, cells were fixed with 4% paraformaldehyde (PFA) and immunolabeled with primary antibodies against CD86 (1:200, 14-0862-85, Thermo Fisher) and Arginase 1 (1:200, sc-166920, Santa Cruz), followed by incubation with secondary antibodies: donkey anti-mouse IgG-568 (1:400, Thermo Fisher, A10037) and chicken anti-rat IgG-647 (1:400, Thermo Fisher, A-21472). Fluorescent images were acquired using the Pico imaging system (Molecular Devices). Cells were classified based on marker expression as follows: CD86⁺/Arginase-1⁻ as M1-like microglia, CD86⁻/Arginase-1⁺ as M2-like microglia, and CD86⁺/Arginase-1⁺ as intermediate microglia.

For flow cytometry analysis, cells were first blocked with 0.5 µg of anti-CD16/CD32 antibody per 1 × 10^6^ cells (14-0161-82, Thermo Fisher, USA) for 10 min to prevent nonspecific binding. Subsequently, cells were stained with the following surface antibodies: 0.25 µg of FITC-conjugated CD11b (101205, BioLegend, USA), 0.25 µg of PerCP-conjugated CD45 (103129, BioLegend, USA), 0.25 µg of PE-conjugated CD86 (159203, BioLegend, USA), and 0.5 µg of APC-conjugated CD206 (141707, BioLegend, USA), all per 1 × 10^6^ cells. Cells were incubated on ice for 1 h. After staining, cells were fixed with 4% PFA and analyzed using the Cytek^®^ Guava^®^ easyCyte^™^ 5HT Base System (Merck Millipore, Germany).

Microglial subtypes were defined based on surface marker expression as follows:

M1-like microglia: CD45⁺CD11b⁺CD86⁺CD206⁻

M2-like microglia: CD45⁺CD11b⁺CD86⁻CD206⁺

Intermediate phenotype: CD45⁺CD11b⁺CD86⁺CD206⁺

Inactivated phenotype: CD45⁺CD11b⁺CD86⁻CD206⁻

For the NFATc2 nuclear translocation assay, cells were immunolabeled with primary antibodies against NFATc2 (1:200, sc-7296, Santa Cruz), CD86 (1:200, 14-0862-85, Thermo Fisher), and Arginase 1 (1:200, GTX109242, GeneTex), followed by secondary antibodies: donkey anti-mouse IgG-488 (1:400, Thermo Fisher, A-21202), donkey anti-rabbit IgG-568 (1:400, Thermo Fisher, A10042), and chicken anti-rat IgG-647 (1:400, Thermo Fisher, A-21472). Imaging was performed using an LSM900 confocal microscope with Airyscan (Zeiss, Germany). The DAPI and NFATc2 fluorescence channels were imported into Fiji/ImageJ (version 1.54), and colocalization analysis was conducted using the Colocalization plugin to calculate the correlation coefficient.

For all immunocytochemistry (in vivo and in vitro) observers were blinded as to treatment groups and controls were carried out by omission of primary antibodies.

### In Vitro phagocytosis assay

For phagocytosis analysis, BV2 cells were seeded at a density of 1 × 10^4^ cells/well on coverslips in 6-well plates. The next day, media were replaced with DMEM containing 2% FBS, and cells were pretreated for 30 min with 1 µM Maraviroc (HY-13004, MedChemExpress), 100 nM SB-328437 (HY-103363, MedChemExpress), or 10 µM BX471 (HY-12080, MedChemExpress). Cells were then treated with 500 pg/ml or 750 pg/ml recombinant CCL5 for 30 min before incubation with 1 µg/mL β-Amyloid-555 (Anaspec, AS-60480–01) for 2 h. Immunostaining was performed with EEA1 (1:200, GeneTex, GTX109638), followed by anti-rabbit-488 (1:400, Thermo Fisher, A-21206). Imaging was carried out on a Stellaris 8 confocal microscope (Leica, Germany).

### Statistical analysis

Data are presented as mean ± SEM and the statistical analysis was conducted using GraphPad Prizm 8.0 (GraphPad Software, Dan Diego, CA, USA). An unpaired t-test was used for comparisons between two groups. One-way ANOVA was performed to analyze the effects of a single factor, while two-way ANOVA was employed to assess the interaction between two factors. A *p* value < 0.05 was considered significant.

## Results

### Impaired inflammatory activation in the cortex of CCL5-KO mice after mild traumatic brain injury.

**Fig. 1:**
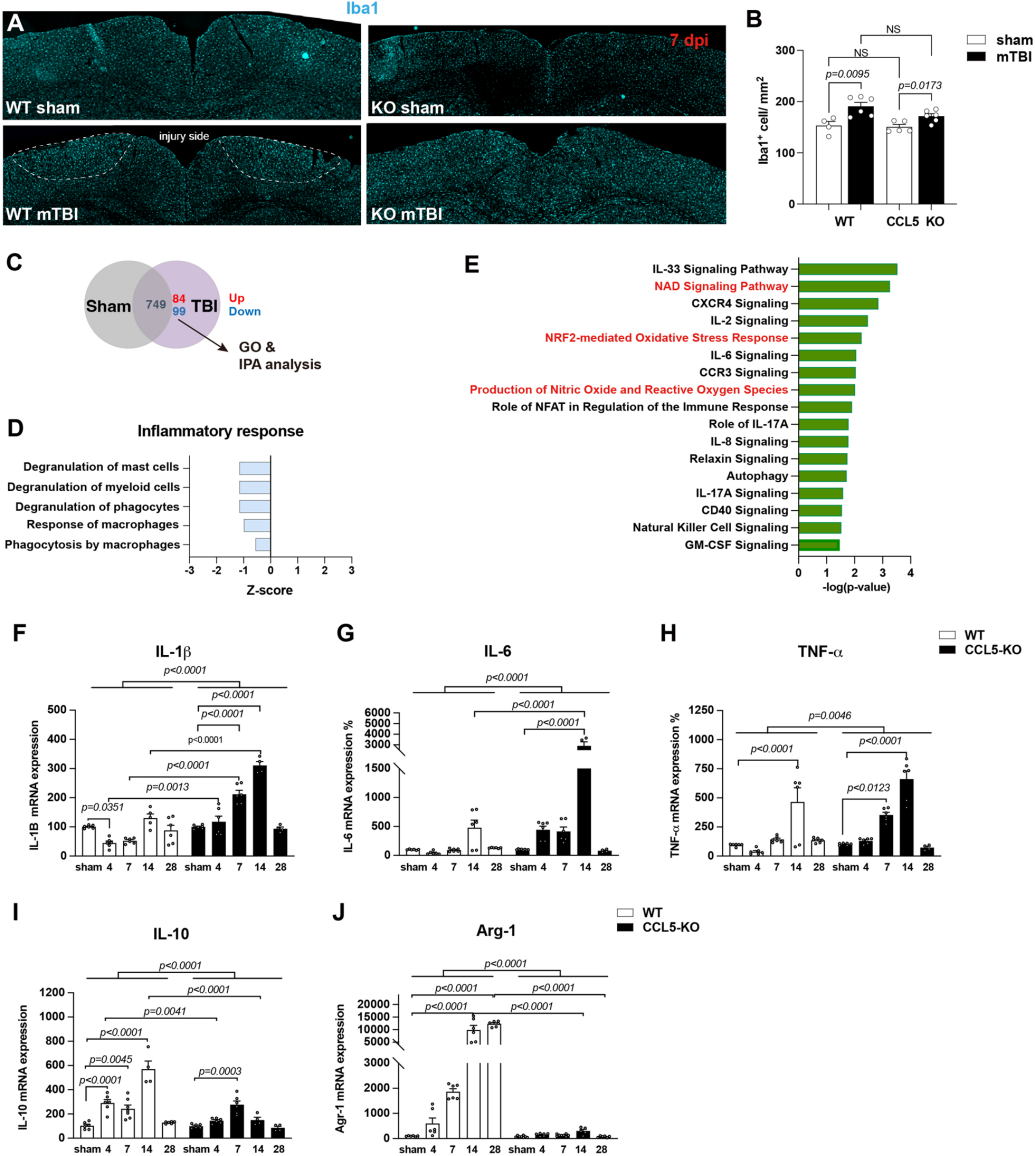
CCL5 regulates oxidative and immune system activation in brain after injury. **A** Microglial accumulation (Iba1⁺, cyan) was observed at the injury site in the cortex of WT and CCL5-KO mice following mild traumatic brain injury (mTBI). Dashed lines outline regions of microglial enrichment. **B** Quantification of Iba1⁺ cells from 4–6 mice per group and analyzed by Mann Whitney test. (Each dot represents the mean value counted from 3 different slides from each single mouse.) NS: no significant difference. **C** Cortical tissues from injured and sham mice were analyzed by LC-MS/MS. Identified proteins were subsequently subjected to Gene Ontology (GO) and Ingenuity Pathway Analysis (IPA). **D** GO analysis revealed alterations in disease and immune/phagocytosis-related functions. *Z*-score values suggest predicted inhibition of these functions (blue bars). **E** IPA identified canonical pathways related to immune–microglial signaling and oxidative stress (highlighted in red). **F**–**H** mRNA expression levels of pro-inflammatory cytokines: TNF-α, IL-1β, and IL-6. **I**–**J** mRNA expression levels of anti-inflammatory markers: IL-10 and Arg-1. Quantitative PCR was performed on cortical tissue from WT and CCL5-KO mice under sham conditions and at 4-, 7-, 14-, and 28-days post-injury. Sham group values were set to 100%, and mTBI data were normalized accordingly. **F**-**J**, data are presented as mean ± S.E.M. and analyzed by Two-Way ANOVA following Tukey’s multiple comparisons test

Mild traumatic brain injury (mTBI) was induced in wild-type (WT, C57BL/6) and CCL5-knockout (CCL5-KO) mice at two months of age using a closed-head weight-drop (WD) model. To examine the impact of CCL5 deletion on inflammatory responses after injury, we performed immunostaining for Iba1, a marker for microglia, in the mouse cortex 7 days after mTBI. An increase in Iba1-positive (Iba1⁺) cells was observed in the cortex of both WT and CCL5-KO mice after mTBI (Fig. 1A, B). In WT mice, Iba1⁺ microglia accumulated prominently at the lesion site (Fig. 1A, circled area), whereas CCL5-KO mice exhibited sparse microglial presence, suggesting impaired recruitment. However, the overall number of Iba1⁺ cells did not differ significantly between WT and CCL5-KO mice in either the sham or mTBI groups (Fig. 1B).

To further investigate inflammatory and oxidative stress status, cortical tissues from sham and mTBI CCL5-KO mice were analyzed one-month post-injury using LC–MS/MS proteomics [23]. Gene Ontology (GO) analysis revealed significant downregulation of key inflammatory pathways in CCL5-KO cortices post-injury, including macrophage-mediated phagocytosis, macrophage activation, and dysregulation of phagocytes, myeloid cells, and mast cells (Fig. 1C, D). Ingenuity Pathway Analysis (IPA) further identified the inflammatory signaling pathways involving chemokines such as IL-33, IL-2, IL-6, IL-17A, and IL-8, as well as oxidative stress-related pathways, including ferroptosis, NAD signaling, NRF2-mediated oxidative stress response, and nitric oxide/reactive oxygen species (NO/ROS) production (Fig. 1E, red character labeled, see also [23]). Interestingly, one receptor for CCL5—CCR3 related signaling was also identified (Fig. 1E).

To validate these findings, we performed quantitative PCR to measure mRNA levels of inflammation-associated cytokines in the cortex of WT and CCL5-KO mice after injury. Expression of pro-inflammatory cytokines—IL-1β, IL-6, and TNF-α—was elevated in both genotypes following mTBI up to 14 days post-injury (dpi), with significantly higher expression in CCL5-KO mice between 4 and 14 dpi (Fig. 1F–H). In contrast, the anti-inflammatory cytokine IL-10 was significantly upregulated in WT mice at 4 to 14 dpi; Arginase-1 (Arg-1) expression was sustained until 28 dpi in WT mice, which was not observed in CCL5-KO mice (Fig. 1I, J).

Despite the elevated expression of pro-inflammatory cytokines in CCL5-KO mice, proteomic profiling indicated a paradoxical suppression of canonical inflammatory signaling pathways, potentially reflecting dysregulated immune homeostasis.

### CCL5 deficiency increased oxidative stress in neurons and microglia after injury.

Proteomic analyses further revealed that CCL5 deficiency affected several oxidative stress-related pathways, including NAD signaling and nitric oxide (NO)/reactive oxygen species (ROS) signaling in the cortex of TBI mice (Fig. 1E).

**Fig. 2:**
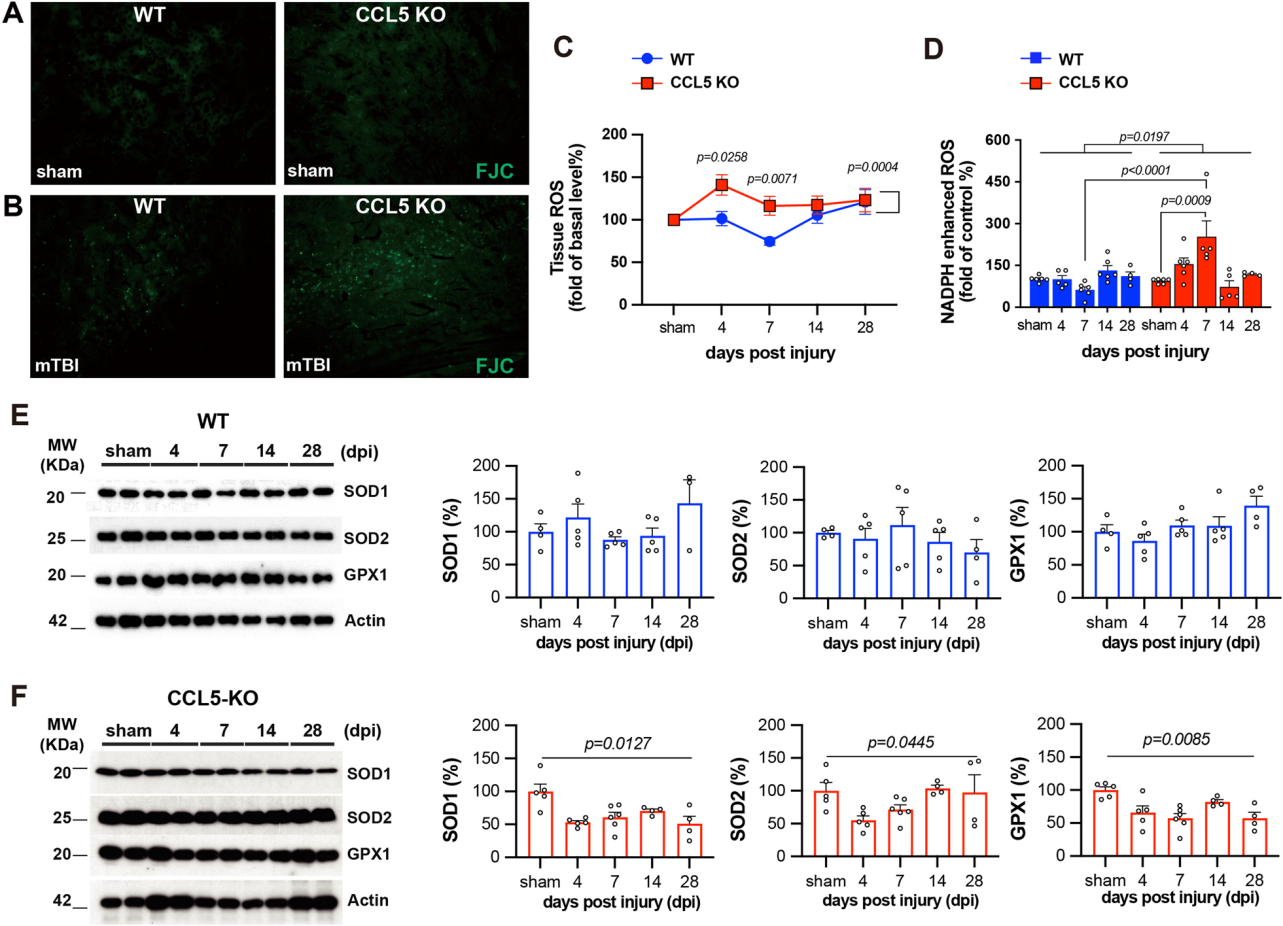
Neuronal damage and oxidative stress in mouse cortex following brain injury. **A**–**B** Degenerating neurons were labeled by Fluoro-Jade C (FJC) staining (green) in the cortex of sham and mTBI-treated WT and CCL5-KO mice. **C** Baseline and post-injury cortical ROS levels were assessed at 4-, 7-, 14-, and 28-days post-injury in WT and CCL5-KO mice.(Data were analyzed by two-way ANOVA following Tukey’s multiple comparisons test; data presented as mean ± S.E.M.; N = 4–8 mice per group, with two replicates per animal.) **D** NADPH oxidase-dependent ROS production in cortical tissue from WT and CCL5-KO mice at the same post-injury time points. (Data were analyzed by two-way ANOVA following Tukey’s multiple comparisons. N = 4–8 mice per group, with two replicates per animal) (**E**–**F**) Expression levels of antioxidant proteins SOD1, SOD2, and GPX1 in cortical tissue of sham and injured mice at 4-, 7-, 14-, and 28-days post-injury. (Data were analyzed by one-way ANOVA; N = 4–6 mice per group.)

To evaluate neuronal damage, we first performed Fluoro-Jade C (FJC) staining. A significant increase in FJC-positive cells was observed in the cortex of CCL5-KO mice at 28 days post-injury (dpi), whereas no such increase was found in WT mice (Fig. 2A, B). To evaluate oxidative stress dynamics, we measured baseline and NADPH oxidase-dependent ROS production in cortical tissue at multiple time points post-injury. Interestingly, ROS levels and NADPH oxidase activity remained largely unchanged in WT mice after TBI (Fig. 2C, D, blue) which suggest a rapid balance of OS in WT mice cortex after impact. In contrast, both ROS and NADPH oxidase activity were significantly elevated in the CCL5-KO cortex during 4 to 7 dpi (Fig. 2C, D, red).

We also examined the protein levels of key antioxidant enzymes—superoxide dismutase 1 (SOD1), superoxide dismutase 2 (SOD2), and GPX1—in cortical tissue. These enzymes are critical for the detoxification of superoxide and hydrogen peroxide, and their downregulation may exacerbate oxidative neuronal injury. In WT mice, the levels of these enzymes did not change significantly at 4, 7, 14, or 28 dpi (Fig. 2E). However, in CCL5-KO mice, levels of SOD1 and GPX1 were markedly reduced following brain injury (Fig. 2F). Taken together, these findings indicate that CCL5 expression is essential for the activation of GPX1 and SOD1 in the cortex after injury.

**Fig. 3:**
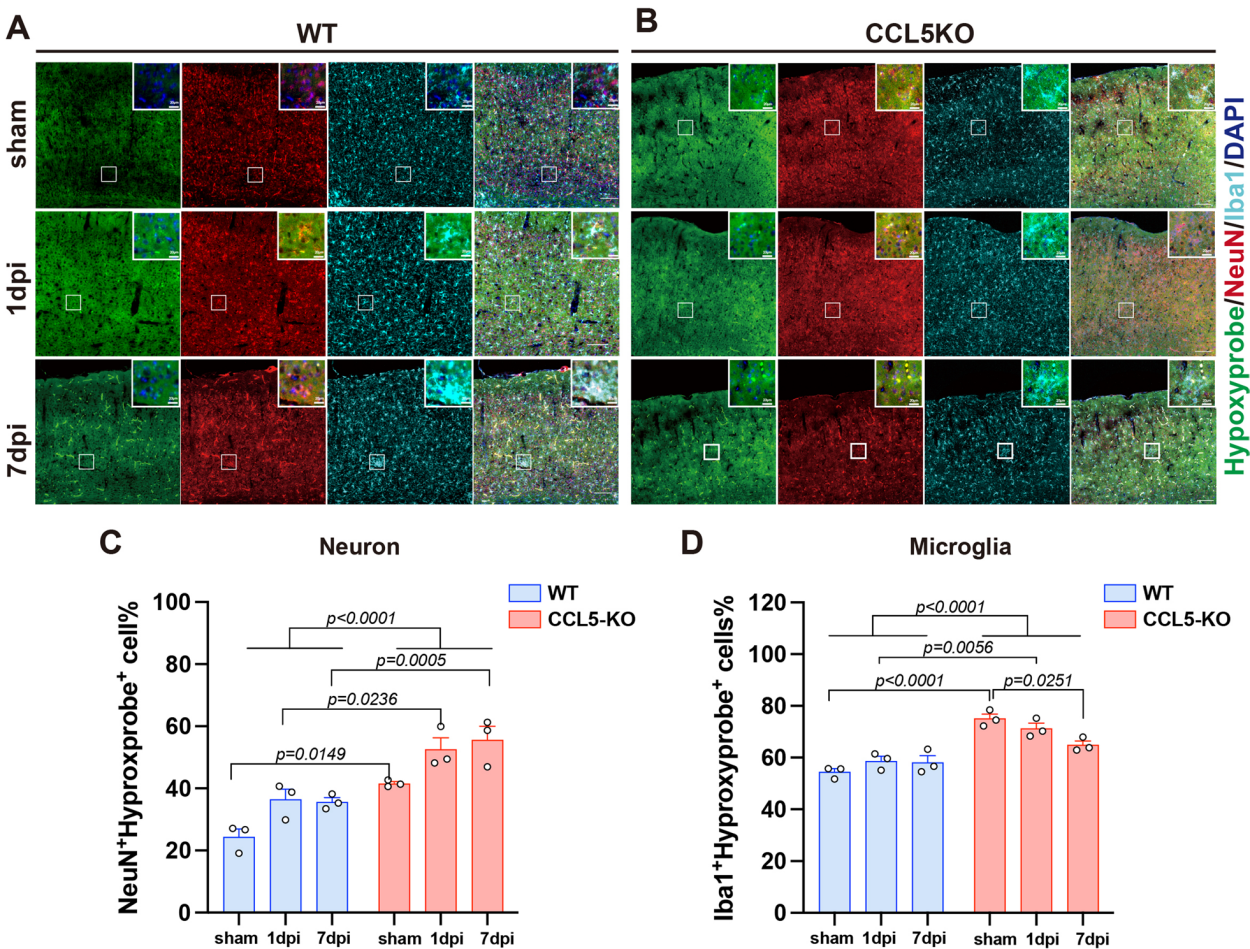
Oxidative stress increases in both neurons and microglia in the cortex of WT and CCL5-KO mice after brain injury. **A**–**B** Cells undergoing oxidative stress were labeled with Hydroxyprobe™-1 (green), with co-labeling of cortical neurons by NeuN (red) and microglia by Iba1 (cyan). Nuclei were counterstained with DAPI (blue). Boxed regions are enlarged in the upper right corner. Scale bar: 100 μm. **C**–**D** Quantification of double-positive cells for Hydroxyprobe™-1 and NeuN (**C**), or Hydroxyprobe™-1 and Iba1 (**D**), normalized to the number of NeuN⁺ or Iba1⁺ cells, respectively. (Data were analyzed by two-way ANOVA following Tukey’s multiple comparisons test. N = 3 mice per group. Each dot represents the mean value counted from 3 different slides from each single mouse.)

To further investigate cell-type-specific oxidative stress, we performed double-label immunofluorescence using Hydroxyprobe^™^-1 (green), a marker of hypoxia-induced oxidative stress, in combination with the neuronal marker NeuN (red) and the microglial marker Iba1 (cyan) (Fig. 3A, B). Oxidative stress in neurons (Hydroxyprobe⁺/NeuN⁺ cells) increased in WT cortices at 1 and 7 dpi, with an even greater increase observed in CCL5-KO mice (Fig. 3C). Oxidative stress in microglia (Hydroxyprobe⁺/Iba1⁺ cells) also increased in WT mice following injury; however, in CCL5-KO mice, microglial oxidative stress was progressively reduced after injury (Fig. 3D). Notably, even under sham conditions, the number of Hydroxyprobe⁺/NeuN⁺ and Hydroxyprobe⁺/Iba1⁺ cells were higher in CCL5-KO mice compared to WT, suggesting that CCL5 is required for the effective clearance of oxidative stress not only in neurons but also in microglia under basal and injury conditions.

### CCL5 protects microglia from oxidative stress-induced cell death and promotes M2-like microglia differentiation

**Fig. 4:**
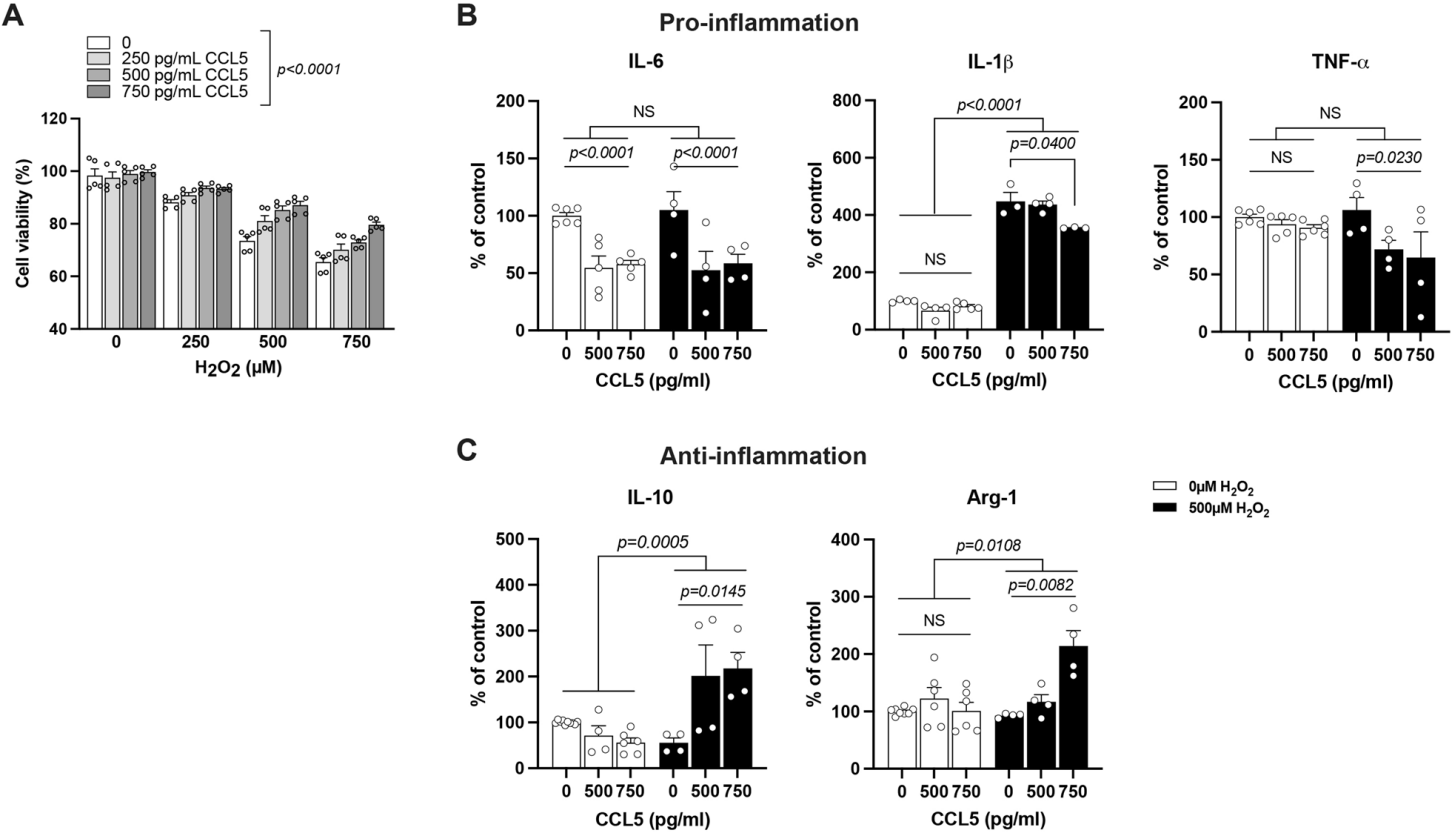
CCL5 promotes microglial survival under oxidative stress and augments M2-like related cytokine expression. **A** BV-2 cells were treated with H_2_O_2_ (0, 250, 500, 1000 µM) to induce oxidative stress. Recombinant CCL5 (0, 250, 500, 750 pg/ml) was administered 30 min prior to H_2_O_2_ exposure. Cell viability was assessed using the MTT assay. CCL5 pretreatment significantly improved BV-2 cell survival under oxidative stress. (Four independent experiments were performed. Statistical analysis by two-way ANOVA.) **B** mRNA expression levels of pro-inflammatory cytokines (IL-6, IL-1β, and TNF-α) and **C** anti-inflammatory cytokines (IL-10 and Arg-1) were measured by qPCR after 6 h of treatment with H_2_O_2_ (500 µM) and CCL5 (0, 500, 700 pg/ml). (Data are presented as mean ± S.E.M. and analyzed by Two-way ANOVA. NS: no significant difference.)

We treated the BV-2 microglial cell line with hydrogen peroxide (H_2_O_2_) to induce cellular oxidative stress and assessed both cell viability and chemokine expression in this in vitro model. Serial concentrations of recombinant CCL5 (0, 250, 500, and 750 pg/mL) were administered with or without co-treatment with H_2_O_2_. Cell viability was measured using the MTT assay. Recombinant CCL5 significantly enhanced the survival of BV-2 microglial cells under oxidative stress, suggesting a cytoprotective role (Fig. 4A).

Exposure to 500 μM H_2_O_2_ markedly increased the expression of pro-inflammatory cytokines IL-6 and IL-1β (Fig. 4B), whereas the expression of anti-inflammatory markers IL-10 and Arg-1 remained largely unchanged (Fig. 4C). In contrast, treatment with CCL5 (500 and 750 pg/mL) significantly upregulated IL-10 and Arg-1 expression under oxidative stress conditions (Fig. 4C). Notably, co-treatment with CCL5 and H₂O₂ significantly suppressed H₂O₂-induced IL-6 and IL-1β expression (Fig. 4B) as well as another inflammatory chemokine- TNF-α, suggesting that CCL5 enhances anti-inflammatory, M2-like microglial responses and attenuates pro-inflammatory activation under oxidative stress.

**Fig. 5:**
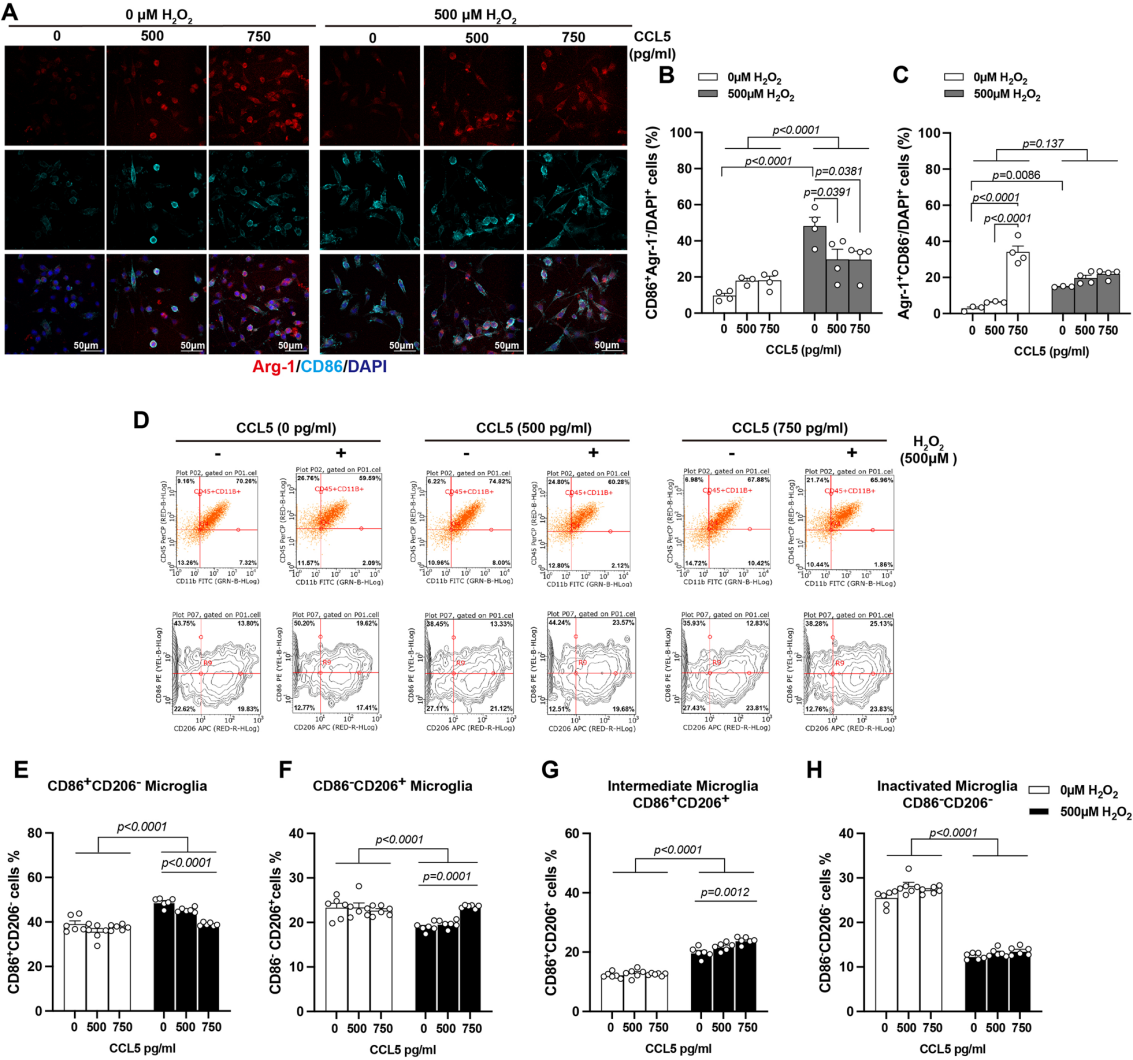
CCL5 reduces oxidative stress-induced M1-like microglia polarization and promotes M2-like microglial differentiation. BV2 cells were treated with H_2_O_2_ (0 or 500 μM) to induce oxidative stress, and co-treated with recombinant CCL5 (0, 500, or 750 pg/mL). **A** Representative immunofluorescence images showing M1-like microglia (CD86⁺, cyan) and M2-like microglia (Arg-1⁺, red) under various treatment conditions. Nuclei were counterstained with DAPI (blue). **B**–**C** Quantification of CD86⁺Arg-1⁻ (M1-like) and CD86⁻Arg-1⁺ (M2-like) cells under different treatments. **D** Microglial phenotypes under oxidative stress and CCL5 treatment were further analyzed by flow cytometry. BV2 cells were “gated” by CD11b and CD45 expression and then subtyped based on CD85 and CD206 markers. **E**–**H** Quantification of microglial subsets: **E** M1-like (CD85⁺CD206⁻), **F** M2-like (CD85⁻CD206⁺), **G** intermediate (CD85⁺CD206⁺), and **H** inactive (CD85⁻CD206⁻) cells. (Data were presented as mean ± S.E.M. and analyzed by Two-way ANOVA following Tukey's multiple comparisons test.)

To further assess microglial polarization, we performed immunostaining for CD86 (a marker of M1-like microglia) and Arg-1 (a marker of M2-like microglia) in BV-2 cells under various treatment conditions. H_2_O_2_ treatment significantly increased both CD86⁺Arg-1^−^ and CD86^−^Arg-1⁺ cell populations (Fig. 5A–C). Interestingly, CCL5 treatment led to a notable increase in CD86^−^Arg-1⁺cells under both control and H_2_O_2_-treated conditions, while it reduced the number of CD86⁺Arg-1^−^ cells in the presence of H_2_O_2_ (Fig. 5A–C).

Flow cytometry analysis confirmed these findings. H₂O₂ exposure induced microglial activation, characterized by an increase in CD86⁺CD206⁺ along with a reduction in CD86⁻CD206⁻ cells (Fig. 5D, G–H). Most activated cells were polarized toward a CD86⁺CD206⁻ (M1-like) phenotype (Fig. 5D, E). While CCL5 alone did not significantly alter CD86 or CD206 expression, co-treatment with CCL5 and H₂O₂ promoted the expansion of CD86⁻CD206⁺ (M2-like) cells and reduced the CD86⁺CD206⁻ population in a dose-dependent manner (Fig. 5D–F).

Taken together, these data suggest that CCL5 not only protects microglia from oxidative stress-induced cell death but also promotes the expression of M2-associated anti-inflammatory genes (IL-10, Arg-1) and facilitates M2-like microglial polarization under oxidative stress conditions.

### Intranasal delivery of recombinant CCL5 after injury reduces oxidative stress and modulates inflammatory chemokine activation

**Fig. 6:**
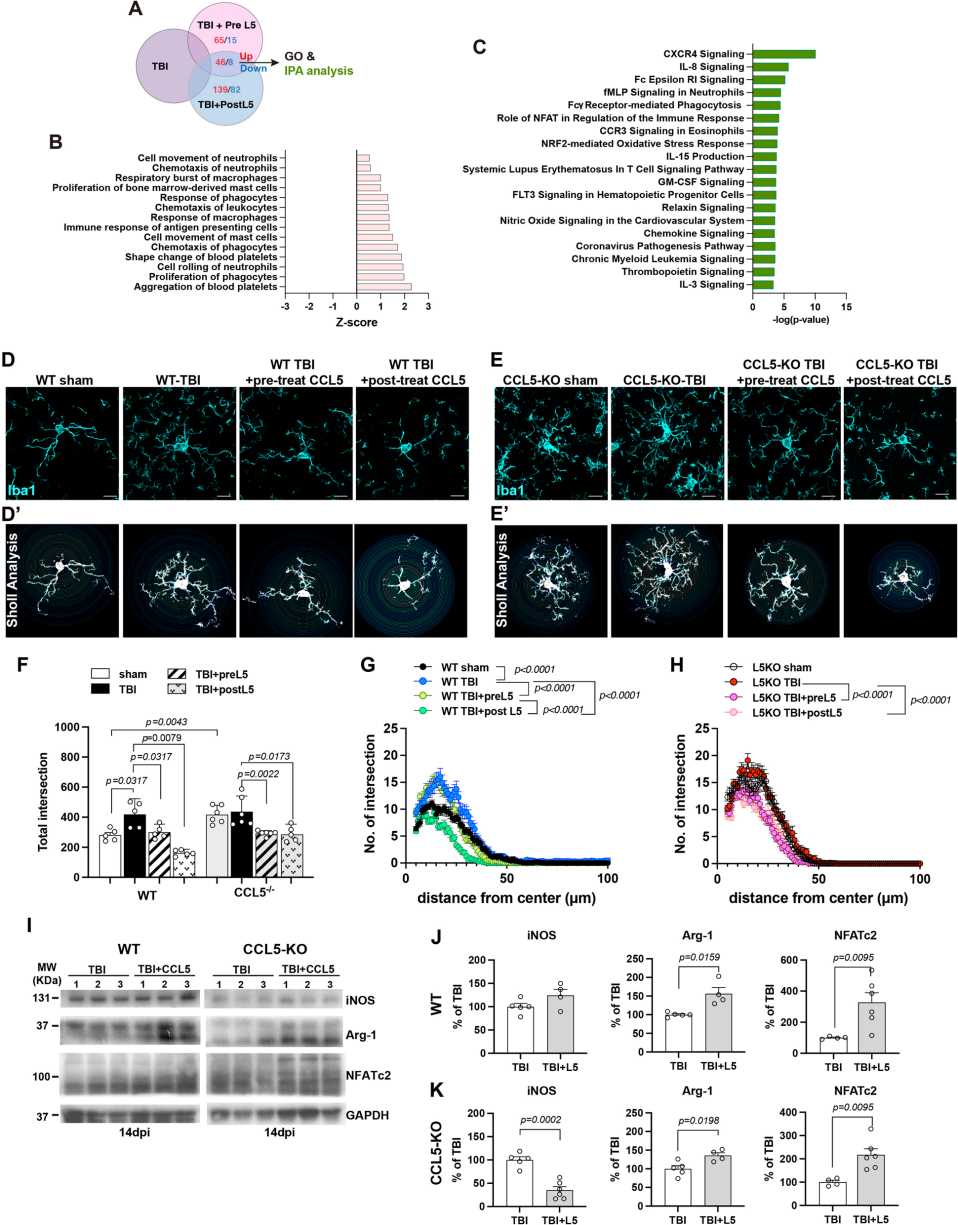
CCL5 administration promotes anti-inflammatory phagocytosis and modulates oxidative status in the injured brain. **A** Cortical tissues from brain injured mice treated with PBS, pre-treated CCL5 or post-treated CCL5 were analyzed by LC-MS/MS. Identified proteins were subsequently subjected to GO and IPA analysis. **B** GO analysis revealed alterations in disease and immune- and phagocytosis-related functions. *Z*-score values indicate predicted activation of these functions (pink bars). **C** IPA identified canonical pathways related to immune–microglial signaling and oxidative stress related signaling. **D**, **E** Microglial morphology after TBI was analyzed by Iba1 immunostaining and Sholl analysis. **F** Total number of intersections and distance-dependent intersection profiles were quantified in WT and CCL5 knockout (CCL5-KO) mice. **G**, **H** Sholl analysis results were further compared between treatment groups in WT (**G**) and CCL5-KO mice (**H**). (Data in **F** were analyzed by Two-way ANOVA following Tukey’s multiple comparisons test; data in **G** and **H** were analyzed by two-way ANOVA. N = 7 per group.) **I** Protein levels of the pro-inflammatory marker iNOS, anti-inflammatory marker Arg-1, and NFATc2 in the cortex were examined in WT and CCL5-KO mice following intranasal administration of PBS or CCL5 after mTBI. **J**, **K** Quantification of protein expression levels. (Data **J**-**K** were analyzed by t-test and are presented as mean ± S.E.M.; *NS* not significant difference; N = 4–6 per group.)

To further investigate the role of CCL5 following brain injury, we administered recombinant CCL5 (300 pg/g) intranasally into the brains of CCL5 knockout (KO) mice immediately prior to the induction of mild traumatic brain injury (mTBI) (Pretreatment CCL5, PreL5, as shown in Supplementary Fig. 3A) or recombinant CCL5 (30 pg/g) from 3 days after injury every 2 days for 28 days (post treatment CCL5, PostL5 as shown in Supplementary Fig. 3A). Intranasal delivery was employed as a non-invasive method to target the brain and bypass the blood–brain barrier that we used in previous studies [23]. Phosphate-buffered saline (PBS) was used as an intranasal control treatment in the CCL5 KO mice undergoing TBI. To evaluate the effects of CCL5 on cortical neurons and microglia, cortical tissues were harvested from CCL5-KO mice subjected to TBI treated with either PBS (TBI control), and recombinant CCL5 (TBI + preL5) or (TBI + postL5) and analyzed via liquid chromatography-tandem mass spectrometry (LC–MS/MS). Gene Ontology (GO) enrichment analysis identified 46 upregulated and 8 downregulated proteins that were consistently altered in both the TBI vs. TBI + preL5 and TBI vs. TBI + postL5 groups (Fig. 6A; adjusted p-value < 0.05). CCL5 treatment enhanced phagocyte proliferation/response, immune cell migration or chemotaxis, and platelet-related functions within the inflammatory response category (Fig. 6B). Ingenuity Pathway Analysis (IPA) further revealed activation of multiple immune-related pathways, including CXCR4 signaling, Fc epsilon RI signaling, fMLP signaling, phagocytosis, NFAT-mediated immune regulation, CCR3 signaling, Relaxin signaling, and chemokine signaling. In addition, CCL5 treatment modulated several oxidative stress-related pathways, such as the NRF2-mediated oxidative stress response, NO signaling, and ferroptosis (Fig. 6C). Analyses comparing TBI vs. TBI + PreL5 and TBI vs. TBI + PostL5 are presented in Supplementary Fig. 3.

Similar rescue experiments were also conducted in WT mice. Microglial activation in the mouse brain was evaluated through Iba1 immunostaining combined with Sholl analysis and protein expression profiling. In WT sham mice, microglia exhibited a homeostatic morphology (Fig. 6D-D’). Interestingly, CCL5-KO sham mice showed a greater number of microglial intersections, suggesting a more reactive microglial state even in the absence of brain injury (Fig. 6E, F). Following TBI, microglial branching increased in WT mice but showed limited change in KO mice (Fig. 6D–F). Both WT and KO mice displayed a hyperramified microglial morphology post-TBI (Fig. 6D–F). Notably, intranasal administration of CCL5 attenuated microglial activation in both WT and KO mice following TBI, with the effect being particularly prominent in the post-treatment groups. (Fig. 6D–H).

At the protein expression level, the pro-inflammatory marker inducible nitric oxide synthase (iNOS) was reduced in KO mice receiving CCL5 post-TBI, while no significant change was observed in WT mice (Fig. 6I–K). Conversely, the anti-inflammatory marker arginase-1 (Arg-1) was upregulated in both WT and KO mice following CCL5 treatment (Fig. 6I–K). These data suggest that exogenous CCL5 can partially rescue the immune and oxidative-positive phenotypes observed in CCL5-KO mice.

### CCR5 mediates CCL5-induced microglial phagocytosis and anti-inflammatory differentiation.

To further validate proteomic findings suggesting increased phagocytic activity following CCL5 administration, we utilized an in vitro BV2 microglial cell culture model. β-amyloid (1–42)-Alexa Fluor 555 was added to the culture medium, and cells were treated with varying concentrations of CCL5 (0, 500, and 750 pg/mL), either alone or in combination with specific inhibitors targeting CCL5 receptors—CCR1, CCR3, and CCR5.

**Fig. 7:**
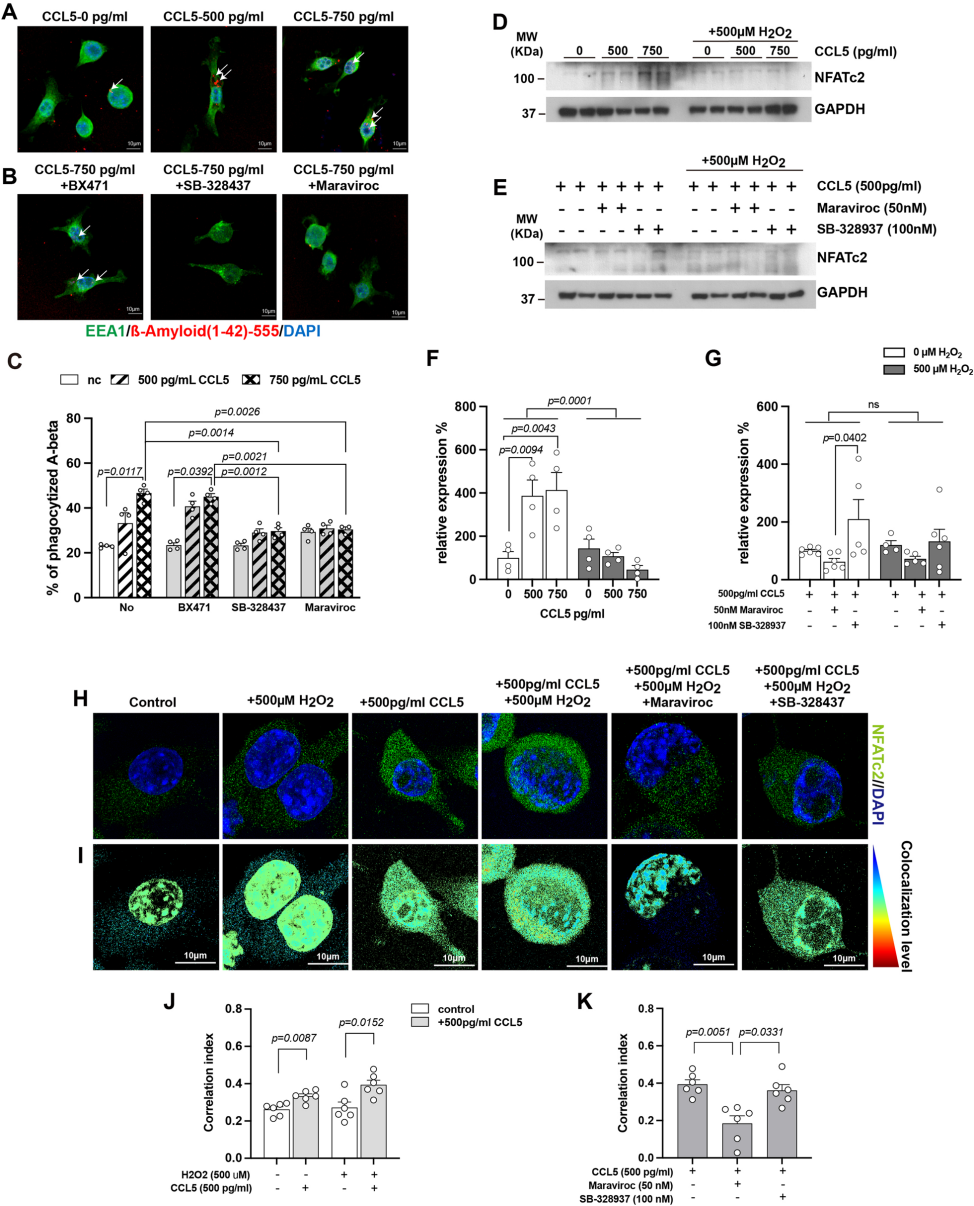
Activation of BV2 cell phagocytosis and NFATc2 in response to CCL5 and its receptor inhibitors. **A**–**B** Phagocytosis of β-amyloid (1–42) was assessed by immunostaining of early endosomes (EEA1, green) and β-amyloid (1–42)-Alexa Fluor 555 (red). **A** CCL5 treatment increased the number of phagocytosed β-amyloid (1–42) particles. **B** The enhanced phagocytic activity induced by CCL5 was inhibited by the CCR3 inhibitor SB328437 (100 pM) and the CCR5 inhibitor Maraviroc (10 nM), but not by the CCR1 inhibitor BX471 (50 nM). Quantification is shown in (**C**). (**C**, data analyzed by Two-way ANOVA following Tukey’s multiple comparisons test. Data are presented as mean ± S.E.M.; n = 4–5 per group). **D**–**E** Protein levels of NFATc2 in BV2 cells were analyzed under treatment with H₂O₂, CCL5, and CCL5 receptor inhibitors. Quantifications are shown in (**F**) and (**G**). (Data in **F**, **G** were analyzed by Two-way ANOVA following Tukey’s multiple comparisons test.) **H**–**I** Subcellular localization of NFATc2 following various treatments visualized by immunostaining (green). **J**-**K** Quantification of nuclear versus cytosolic NFATc2 localization. (n = 4–5 per group.) (Data in **J** were analyzed by Mann Whitney test; Data in **K** were analyzed by Kruskal-Wallis test following Dunn’s multiple comparisons test.) Nuclei were counterstained with DAPI (blue). Scale bars: 10 μm in **A**–**B**; 5 μm in **H**–**I**

Phagocytosis of β-amyloid was evaluated via immunostaining using the early endosome marker EEA1 (green) and β-amyloid (1–42)-Alexa-555. CCL5 treatment significantly enhanced phagocytic activity in a dose-dependent manner (Fig. 7A, C, Supplementary Fig. 2A). This enhancement was effectively blocked by the CCR3 inhibitor SB328437 (100 pM) and the CCR5 inhibitor Maraviroc (10 nM) (Fig. 7B, C, Supplementary Fig. 2A), but not by the CCR1 inhibitor BX471 (50 nM), indicating that CCR3 and CCR5—but not CCR1—mediate the CCL5-induced enhancement of microglial phagocytosis. CCL5 treatment upregulated CCR3 expression in BV2 cells (Supplementary Fig. 1A), but not CCR5 (Supplementary Fig. 1B). Notably, co-treatment with CCL5 and H_2_O_2_ increased the expression of both CCR3 and CCR5 (Supplementary Fig. 1A-B).

Proteomic analysis identified NFATc2, a key regulator of immune function, as a downstream target (Fig. 6C). NFATc2 activation involves dephosphorylation and nuclear translocation. We further examined the relationship between CCL5 signaling and NFATc2 activation. CCL5 treatment increased cytosolic NFATc2 expression (Fig. 7D, F), whereas H₂O₂ treatment reduced it. Immunofluorescence staining confirmed changes in NFATc2 localization (Fig. 7H–J; Supplementary Fig. 2B). Interestingly, CCL5 alone did not induce NFATc2 nuclear translocation, but H_2_O_2_ alone or co-treatment with CCL5 did promote nuclear translocation (Fig. 7H–J; Supplementary Fig. 2B).

The increase in cytosolic NFATc2 expression induced by CCL5 was significantly attenuated by the CCR5 inhibitor Maraviroc, but not by the CCR3 inhibitor SB328437 (Fig. 7E, G). Maraviroc also reduced NFATc2 nuclear translocation (Fig. 7H, I, K), suggesting a specific role for CCR5 in this signaling pathway. Collectively, these results suggest that CCL5 and H_2_O_2_ differentially modulate NFATc2-mediated immune responses via distinct CCL5 receptor pathways—primarily through CCR5 and CCR3.

### CCR5 is the primary receptor mediating CCL5-induced M2-like microglia differentiation

**Fig. 8:**
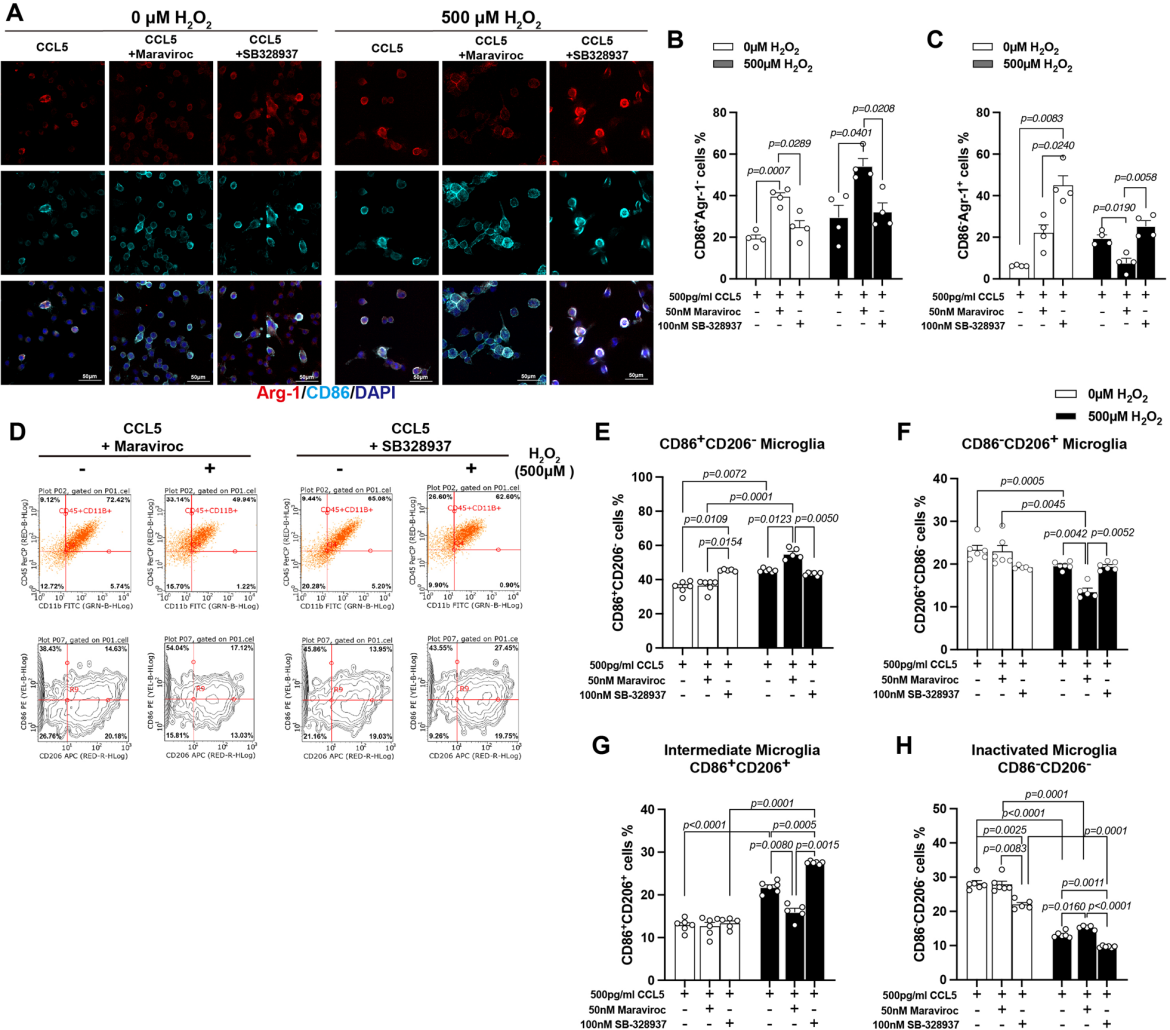
Blocking the CCR5 receptor abolishes CCL5-mediated M2-like microglial differentiation. BV2 cells were treated with H_2_O_2_ (0 or 500 μM), recombinant CCL5 (500 pg/mL), and either the CCR5 inhibitor Maraviroc (50 nM) or the CCR3 inhibitor SB328437 (100 nM). **A** Representative immunofluorescence images showing M1-like microglia (CD86⁺, cyan) and M2-like microglia (Arg-1⁺, red) under different treatment conditions. Nuclei were counterstained with DAPI (blue). **B**, **C** Quantification of CD86⁺Arg-1⁻ (M1-like) and CD86⁻Arg-1⁺ (M2-like) cells under each condition. (Data were analyzed by Kruskal–Wallis test following Dunn’s multiple comparisons test and presented as mean ± S.E.M.) **D** BV2 cell populations were further analyzed by flow cytometry. Cells were first “gated” by CD11b and CD45 expression, then subtyped based on CD85 and CD206 expression. **E**–**H** Quantification of microglial subtypes: **E** M1-like (CD85⁺CD206⁻), **F** M2-like (CD85⁻CD206⁺), **G** intermediate (CD85⁺CD206⁺), and **H** inactive (CD85⁻CD206⁻) microglia. (Data were analyzed by Two-way ANOVA following Tukey’s multiple comparisons test and presented as mean ± S.E.M.)

To investigate the roles of CCR3 and CCR5 in microglial polarization, we performed immunostaining for CD86 and Arg-1 in BV2 cells treated with CCL5 (500 pg/mL) in the presence or absence of Maraviroc (50 nM, CCR5 inhibitor) and SB328437 (100 nM, CCR3 inhibitor), under both normal and oxidative stress (H_2_O_2_-treated) conditions. Inhibition of CCR5 with Maraviroc significantly increased the proportion of CD86⁺Arg1⁻ (M1-like) BV2 cells under both conditions (Fig. 8A, B). Interestingly, inhibition of CCR3 or CCR5 increased CD86⁻Arg1⁺ (M2-like) cells under normal conditions, but CCR5 inhibition reduced the M2-like population under co-treatment with CCL5 and H_2_O_2_ (Fig. 8A, C).

Flow cytometric analysis supported these findings. CCR3 or CCR5 inhibition reduced overall BV2 activation (Fig. 8D, H), increased the M1-like (CD86⁺Arg1⁻) population (Fig. 8D, E), and decreased the M2-like (CD86⁻Arg1⁺) population (Fig. 8D, F).

Among the three receptors, CCR5 appears to be the primary mediator of CCL5-induced NFATc2 activation and subsequent M2-like microglial differentiation under oxidative stress. Given that CCR5 is pharmacologically targetable (e.g., Maraviroc), these findings have potential translational relevance.

### CCL5 administration attenuates neuronal and microglial oxidative stress following injury

**Fig. 9:**
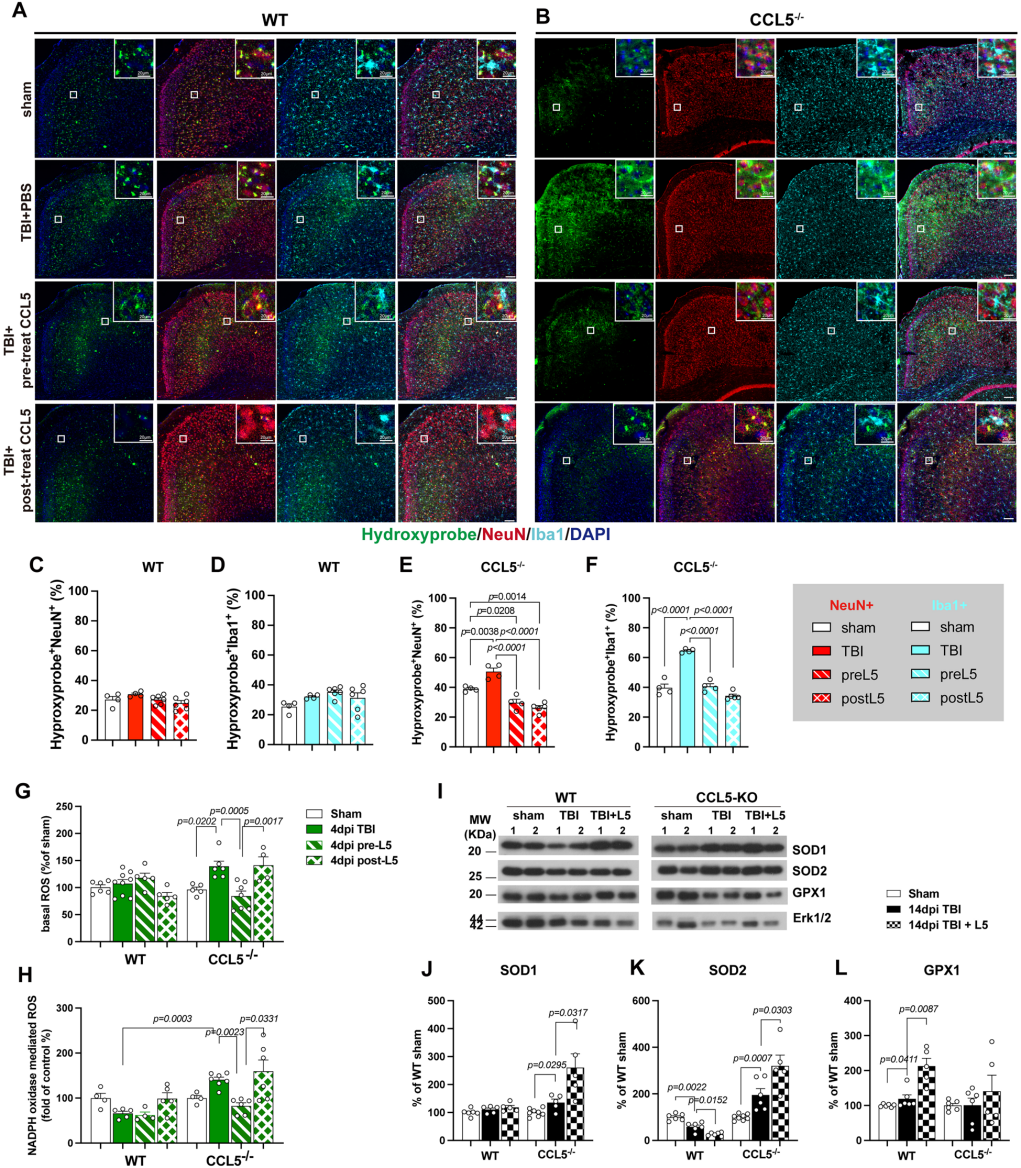
CCL5 administration reduces oxidative stress in the mouse brain after mild traumatic brain injury (mTBI). **A, B** Oxidative stress was detected using Hydroxyprobe™-1 (green), with co-labeling for neuronal marker NeuN (red) and microglial marker Iba1 (cyan). Nuclei were counterstained with DAPI (blue). Boxed regions indicate areas of colocalization of Hydroxyprobe™-1 with NeuN or Iba1, and are shown in higher magnification in the upper right corner. Scale bar: 100 μm and 20 μm. **C**–**F** Quantification of double-positive cells for Hydroxyprobe™-1 and NeuN (**C**, **E**), or Hydroxyprobe™-1 and Iba1 (**D**, **F**), normalized to the number of NeuN⁺ or Iba1⁺ cells, respectively. (Data were analyzed using One-way ANOVA followed by multiple comparisons test and are presented as mean ± S.E.M.) Each dot represents the mean value obtained from one mouse. **G**, **H** Basal ROS levels and cortical ROS production after mTBI were measured by NADPH oxidase activity. CCL5 treatment significantly reduced tissue ROS levels. (Data were analyzed using Two-way ANOVA followed by Tukey’s multiple comparisons test and are presented as mean ± S.E.M.) **I** Protein expression levels of antioxidant enzymes, SOD1, SOD2, and GPX in the cortex of wild-type (WT) and CCL5 knockout (CCL5-KO) mice. **J**-**L** Quantification of antioxidant protein levels. Data were analyzed by Kruskal-Wallis test following Dunn’s multiple comparisons test and are presented as mean ± S.E.M. (n = 5–8 animals per group.)

To assess the effects of CCL5 on oxidative stress in the injured brain, Hydroxyprobe™ labeling was used, in conjunction with NeuN (neuronal marker) and Iba1 (microglial marker) immunostaining, to evaluate cellular oxidative stress in the cortices of WT and CCL5-KO mice. Comparisons were made between sham-operated CCL5-KO mice, CCL5-KO mice receiving PBS, and those treated with CCL5 following mild traumatic brain injury (mTBI) (Fig. 9A). Quantitative analysis revealed that CCL5 administration significantly reduced oxidative stress in both neurons and microglia after mTBI (Fig. 9B, C).

The cellular OS showed minimal changes in WT mice (Fig. 9A, C–D). However, both CCL5 pretreatment and posttreatment significantly reduced oxidative stress levels in neurons and microglia induced by TBI (Fig. 9B, E-F).

CCL5 pre-treatment rapidly reduced cortical ROS levels generated by NADPH oxidase in CCL5-KO mice as early as 4 days post-injury (dpi). In contrast, CCL5 post-treatment did not produce a comparable effect after a 1-day treatment period (post-treatment initiated at 3 dpi, with ROS levels and NADPH oxidase induction assessed at 4 dpi) (Fig. 9G-H). In WT mice, neither basal ROS levels nor NADPH-oxidase–induced ROS were altered by CCL5 administration (Fig. 9G-H). Among antioxidant enzymes, glutathione peroxidase 1 (GPX1) was upregulated after mTBI in wild-type (WT) mice and was further elevated, approximately two-fold, by CCL5 treatment (Fig. 9I, L). In contrast, superoxide dismutase isoforms SOD1 and SOD2 were not significantly upregulated by injury alone but were markedly increased upon CCL5 administration in CCL5-KO injured brain tissue (Fig. 9I-K).

In summary, our results demonstrate a dual role for CCL5 in modulating immune responses and mitigating oxidative stress following mTBI. Mechanistically, these effects are mediated predominantly through CCR5-dependent signaling in microglia, suggesting a novel therapeutic axis for post-TBI intervention.

## Discussion

In the present study, we found that CCL5 plays a pivotal role in the elimination of oxidative stress, the polarization of microglia toward the M2 phenotype, and the regulation of microglial migration to the injury site by activating SOD1 and NFATc2. Elevated CCL5 expression enhances M2 microglial phagocytic activity and anti-inflammatory functions in the mouse cortex following mTBI, which is positively associated with cortical neuronal functional recovery, as also demonstrated in our previous study [23]. Pharmacological inhibition of the CCL5 receptor CCR5 attenuated both CCL5- and H₂O₂-induced M2 microglial polarization and phagocytic function.

We previously identified reduced antioxidant activity in hippocampal neurons of CCL5-KO mice, and demonstrated that CCL5-mediated activation of GPX1 expression is essential for reactive oxygen species (ROS) reduction in hippocampal neurons [22]. In the present study, we found that cortical tissue in WT mice exhibits higher antioxidant activity following mTBI (Fig. 2C–D). Unlike the hippocampus, ROS generated in the cortex of WT mice was efficiently eliminated without significant upregulation of antioxidant proteins such as SOD1, SOD2, and GPX1 (Fig. 2E). In contrast, this clearance of ROS was impaired in CCL5-KO mice (Fig. 2C-D). Notably, the protein levels of SOD1 and GPX1 were significantly reduced in the cortex of CCL5-KO mice post-injury, accompanied by elevated ROS levels (Fig. 2D, F), indicating compromised antioxidant defenses in the absence of CCL5. These findings suggest that CCL5 is required for the activation of SOD1 and GPX1 in cortical tissue following mTBI. Furthermore, intranasal administration of recombinant CCL5 increased SOD1 and SOD2 protein expression in the cortex of CCL5-KO mice (Fig. 9I-K), further supporting the role of CCL5 in regulating antioxidant responses. The association between SOD1 (Cu^2^⁺/Zn^2^⁺-dependent superoxide dismutase) and CCL5 in the nervous system has not been reported; to date, such a relationship has only been identified in the context of hepatitis [31]. Interestingly, we observed elevated ROS levels in both NeuN-positive neurons (Hydroxyprobe⁺/NeuN⁺) and Iba1-positive microglia (Hydroxyprobe⁺/Iba1⁺) in CCL5-KO mice (Fig. 3B-D; Fig. 9B, E-F), suggesting that CCL5 contributes to ROS homeostasis in both neuronal and microglial cell populations.

Furthermore, our previous studies demonstrated that CCL5-KO mice exhibited delayed recovery in motor coordination, sensory sensitivity and cognitive function following mTBI, underscoring the critical role of CCL5 in neurorepair mechanisms [22, 23]. Microglia function is essential for clearing cellular debris, and promoting axonal repair after injury. In the present study, we found that microglial polarization is differentially regulated by H₂O₂-induced ROS and CCL5. Exposure to H₂O₂ promoted M1-like microglial activation, characterized by increased expression of pro-inflammatory cytokines such as IL-1β, IL-6, and TNF-α, without upregulating M2-associated markers IL-10 and Arg-1, both in injured cortical tissue and in cultured BV2 microglial cells (Fig. 1F–J, Fig. 4B-C). Notably, CCL5 alone did not suppress the expression of pro-inflammatory cytokines (IL-1β, IL-6, TNF-α) nor did it significantly upregulate anti-inflammatory markers (IL-10, Arg-1) (Fig. 4B–C). However, when BV2 cells were co-treated with H₂O₂ and CCL5, CCL5 effectively attenuated the H_2_O_2_-induced upregulation of pro-inflammatory cytokines and simultaneously enhanced the expression of IL-10 and Arg-1 (Fig. 4C). In vivo, CCL5 administration following TBI promoted the shift of microglia toward a resting or M2-like phenotype in the mouse cortex, as indicated by reduced branching (Sholl analysis), increased Arg-1 expression, and decreased iNOS levels in Western blot (Fig. 6E–K). Flow cytometry analyses further supported these findings (Fig. 5, 8). Collectively, these data suggest that CCL5 exerts a context-dependent modulatory effect on microglia, particularly under oxidative stress conditions.

Importantly, we observed increased activation of autophagy signaling pathways, phagosome maturation, and endocytosis-related pathways following CCL5 administration, compared to TBI untreated CCL5-KO mice (Fig. 6C). Moreover, CCL5 dose-dependently enhanced β-amyloid phagocytosis (Fig. 7A, C), supporting the notion that M2-like microglial functions are dependent on CCL5. This differential immune modulation between H_2_O_2_ stimulation and the CCL5/CCR5 axis signaling may be mediated through the regulation of NFATc2. NFATc2, a member of the NFAT (nuclear factor of activated T cells) family, is a critical transcription factor involved in the regulation of immune-related genes and chemokine expression. It resides in the cytoplasm in a phosphorylated state and translocates to the nucleus upon dephosphorylation to initiate gene transcription. The potential link between CCL5 and NFAT signaling, seen here, has not been previously investigated. A prior study reported that inhibition of NFAT using INCA-6 reduced IL-1β-induced production of inflammatory chemokines such as IL-1β and TNFα, but not CCL2 or CCL5, in human Müller cells [32]. Here, we demonstrate that CCL5 treatment not only increases cytosolic NFATc2 expression in a dose-dependent manner but also promotes its nuclear translocation (Fig. 7D, F, H–J). Notably, CCR5 plays a critical role in mediating this translocation; NFATc2 nuclear localization induced by combined H_2_O_2_ and CCL5 treatment was attenuated by the CCR5 inhibitor Maraviroc (Fig. 7G, H-I, K). CCR5-dependent NFATc2 nuclear translocation has previously been shown to regulate chemokine expression in T cells [33]; in our study, it was also essential for promoting M2-like microglial polarization and the expression of associated surface markers. In contrast, CCR3, another receptor for CCL5, was not involved in NFATc2 nuclear translocation or the transition of resting microglia (Fig. 7).

Our finding is different from those in other studies. There are many studies using the CCR5 antagonist-Maraviroc in TBI and stroke animal models. The immune response and microglial behavior are profoundly influenced by the specific disease model employed in various studies. In our study, a mild TBI model induced by weight drop was used, characterized by a localized and relatively subtle mechanical impact. This model mimics transient and repairable (CGS13-15) brain injury, where promoting microglial clearance and tissue repair, particularly through M2-like microglial activation, is critical for recovery. In contrast, many previous studies utilizing CCR5 inhibitors have employed models of moderate to severe TBI (< CGS12), ischemic stroke, or chronic neurodegenerative diseases such as Alzheimer’s disease (AD) [24, 26, 34–39]. These conditions often involve systemic or chronic inflammation, where excessive microglial activation contributes to sustained neurotoxicity. Consequently, while CCR5 activation in mild TBI may facilitate M2 microglial polarization and neurorepair, in chronic neurodegenerative conditions, persistent CCR5 signaling has been associated with chronic inflammation and microglial overactivation.

CCR5 appears to be temporally dynamic and dose-dependent, exhibiting potentially opposing effects at different stages of injury or disease progression. CCL5/CCR5 axis activation can support microglial M2 polarization and enhance phagocytic clearance of cellular debris, thereby mitigating oxidative stress and facilitating resolution of inflammation. Lack of CCR5 in the brain leads to severe brain damage after stroke [40]. However, prolonged or excessive CCL5/CCR5 activation in moderate to severe TBI may promote sustained M1-like responses, leading to neuroinflammation and neurotoxicity. Indeed, studies have demonstrated that CCR5 inhibition in moderate to severe TBI and long-term inflammation such as in AD or stroke, can reduce persistent inflammatory signaling. Our previous study also demonstrated a delayed recovery of axon regeneration and telencephalic neuron behavioral functions including rotarod, balance and sensory neglect in CCL5-KO mice. CCL5 administration facilitated axon regeneration and remyelination in both WT and CCL5-KO mice [23]. These phenotypes are consistent with the immune status discovered by q-PCR and LC–MS/MS in current study. CCL5 enhances phagocytosis and repair process. Nevertheless, inhibition of CCR5 may interfere with the initiation of repair mechanisms and impair functional recovery.

This study has some limitations. First, although BV-2 cells are widely used for in vitro studies of microglia, their characteristics are not entirely representative. While they share several properties with primary microglia, such as inflammatory cytokine secretion and phagocytic activity, important differences remain and should be taken into account. Second, the architecture of the mouse lissencephalic, telencephalon, and skull structures are significantly different from the human gyrencephalic, telencephalon. Thus, the effects of WD in models of mouse concussive TBI cannot be fully generalized to human mild (CGS13-15) concussive brain injury.

The dual role of CCL5/CCR5 is also influenced by the surrounding cytokine milieu, oxidative stress levels, and cell-type-specific expression patterns, highlighting the context-dependent nature of CCR5 function. Thus, our findings underscore the importance of temporal and spatial specificity in targeting the CCL5/CCR5 axis. CCL5/CCR5 activation may however promote tissue repair and functional recovery. These results suggest that therapeutic strategies targeting CCL5/CCR5 should consider injury type, phase, and microglial polarization status to optimize efficacy.

## Conclusion

Our findings demonstrate that CCL5 plays a critical role in orchestrating microglial responses following mild traumatic brain injury. By promoting M2-like polarization, enhancing microglial survival, and attenuating oxidative stress, CCL5 contributes to neuroimmune homeostasis in both in vivo and in vitro models. The involvement of CCR5 in mediating CCL5-induced phagocytosis and NFATc2 signaling further underscores the therapeutic potential of targeting the CCL5–CCR5 axis. Collectively, these results suggest that exogenous CCL5 administration may represent a promising strategy to mitigate neuroinflammation and improve functional outcomes after mTBI.

## Supplementary Information

Supplementary material 1.

## Data Availability

The data that support the findings of this study are available from the corresponding author upon reasonable request.

## Abbreviations

Arg-1: Arginase 1
BBB: Blood–brain barrier
CCL5: Chemokine (C-C motif) ligand 5
CCR: C-C chemokine receptor
DAPI: 4’,6-Diamidino-2-phenylindole
Dpi: Days post injury
FITC: Fluorescein isothiocyanate
FJC staining: Fluoro-Jade C staining
GPX-1: Glutathione peroxidase-1
Iba-1: Ionized calcium-binding adapter molecule 1
IL-1β: Interleukin-1β
IL-10: Interleukin-10
IL-6: Interleukin-6
mTBI: Mild traumatic brain injury
NADPH oxidase: Nicotinamide adenine dinucleotide phosphate oxidase
NFATc2: Nuclear factor of activated T-cells, cytoplasmic 2
OS: Oxidative stress
ROS: Reactive oxygen species
SOD: Superoxide dismutase
TNF-α: Tumor necrosis factor-alpha
TBI: Traumatic brain injury
WT: Wild type
i.n.: Intranasal
