# Supplementary material for: CCL5/RANTES signaling in inflammation dysregulation after mild traumatic brain injury
Source: J Biomed Sci. 2026 Jan 9;33:10. doi: 10.1186/s12929-025-01203-0 (PMC12784600; doi:10.1186/s12929-025-01203-0)
Supplement: Supplementary file 1 — Supplementary material 1. [file 12929_2025_1203_MOESM1_ESM.pdf]

**Title: CCL5/RANTES signaling in inflammation dysregulation after mild traumatic brain injury**

Man-Hau Ho[a,b†], Yih-Jeng Tsai[c,d†], Yu-Hsuan Lee[a,b,e,f], Yi-Chen Hsieh[a,b], Chia-Hung Yen[g], Jia-Yi Wang[h,i,j], Thierry Burnouf[j,k,l,m,n], Chia-Yen Chen[b], Wen-Cheng Lin[b], Yun Wang[o], Yung-Hsiao Chiang[i,j,p], Barry J Hoffer[a,b,q,r], Szu-Yi Chou[a,b,j,m,s*]

* Corresponding author

**Supplementary Information:**

**Includes 3 Supplementary Figure legends, 3 Supplementary Figures**

**Supplementary Figures:**

**Figure Legends:**

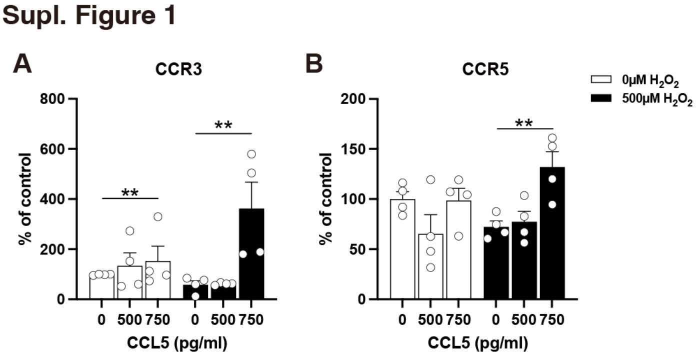


**Supplementary Figure 1: Activation of CCL5-related receptors in BV-2 cells following various treatments.**

The mRNA expression levels of CCL5 receptors CCR3 (A) and CCR5 (B) were measured in BV-2 cells after treatment with $H_2O_2$, CCL5, or a combination of $H_2O_2$ and CCL5.

(Data are presented as mean ± S.E.M. **, $p < 0.01$, analyzed by one-way ANOVA within each treatment group.)

Supl.Figure 2

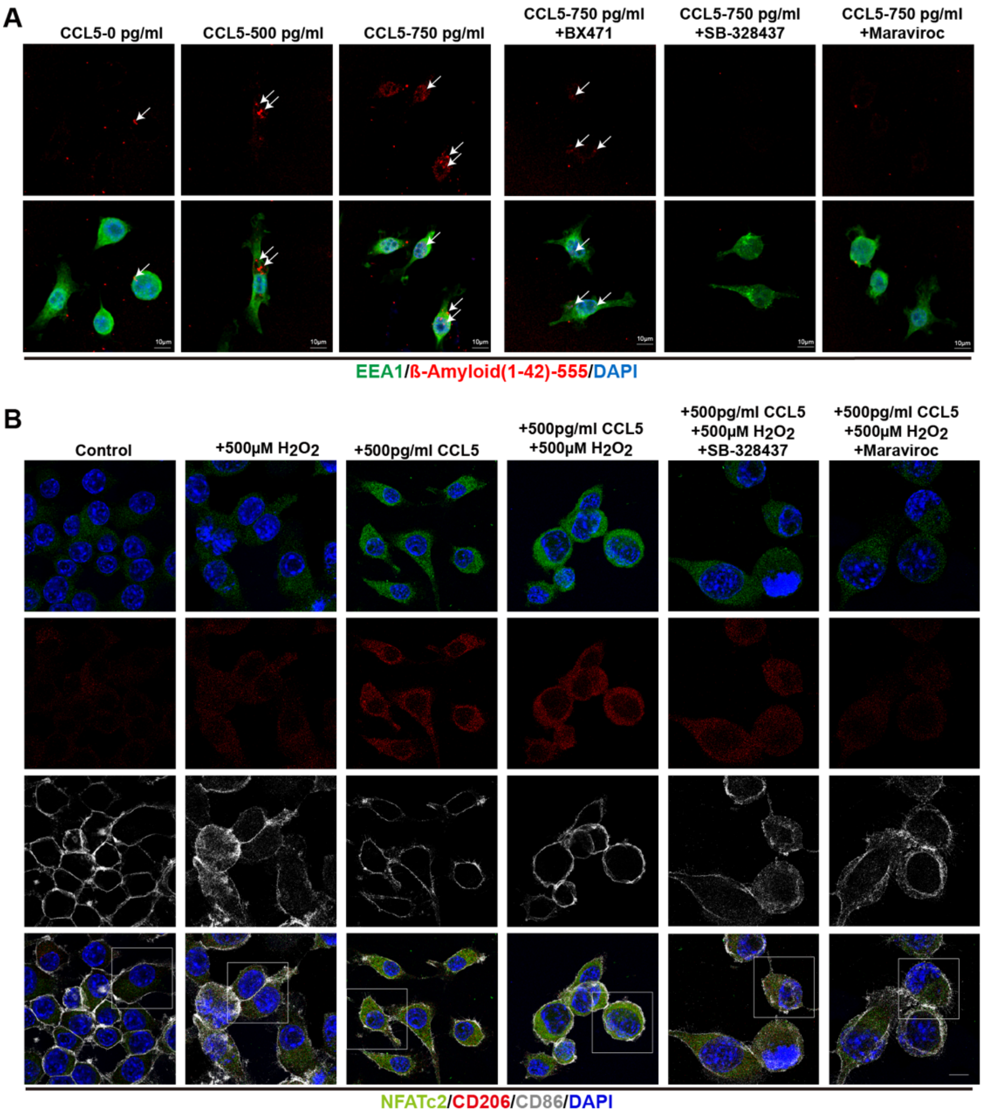


**Supplementary Figure 2: Activation of BV2 cell phagocytosis and NFATc2 in response to CCL5 and its receptor inhibitors.**

(A) Phagocytosis of β-amyloid (1–42) was assessed by immunostaining for early endosomes (EEA1, green) and β-amyloid (1–42)-Alexa Fluor 555 (red).

(B) Cellular distribution of NFATc2 under different treatments was visualized using a specific antibody (green). Microglial markers CD206 and CD86 were labeled with antibodies shown in red and cyan, respectively. White-box regions were showed in Figures 7H-I.

Nuclei were counterstained with DAPI (blue).

Scale bars in (A) and (B), 10 μm.

Supl.Figure 3

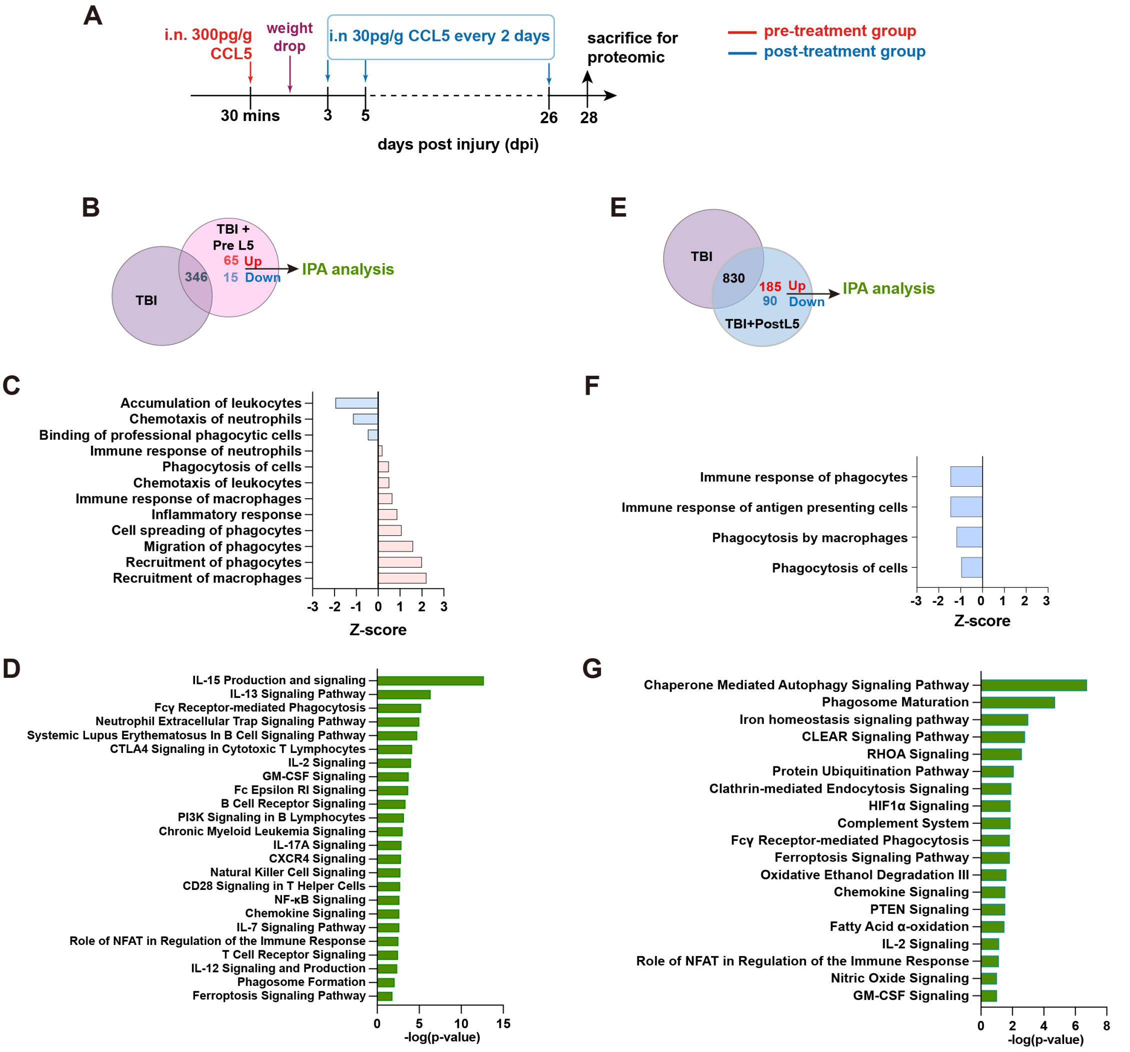


**Supplementary Figure 3. Protein pathway analysis of cortical tissue from CCL5-knockout (CCL5-KO) mice after traumatic brain injury (TBI) with CCL5 pre-treatment or post-treatment.**

(A) Schematic illustration of recombinant CCL5 treatment in TBI mice. (B, E) Venn diagrams showing the comparison of differentially expressed proteins (DEPs) between TBI and TBI with CCL5 pre-treatment (Pre L5) (B), and between TBI and TBI with CCL5 post-treatment (Post L5) (E) in the cortex of CCL5-KO mice.

(C, F) Immune-related categories identified by Ingenuity Pathway Analysis (IPA) in TBI versus TBI with CCL5 pre-treatment (Pre L5) (C), and in TBI versus TBI with CCL5 post-

treatment (Post L5) (F). Z-score values indicate predicted activation (red) or inhibition (blue) of functions.

(D, G) Canonical pathways related to immune and oxidative stress responses identified by IPA in TBI versus TBI with CCL5 pre-treatment (Pre L5) (D), and in TBI versus TBI with CCL5 post-treatment (Post L5) (G).
